# Necroptosis in Pulmonary Diseases: A New Therapeutic Target

**DOI:** 10.3389/fphar.2021.737129

**Published:** 2021-09-14

**Authors:** Lingling Wang, Ling Zhou, Yuhao Zhou, Lu Liu, Weiling Jiang, Huojun Zhang, Huiguo Liu

**Affiliations:** Department of Respiratory and Critical Care Medicine, Key Laboratory of Pulmonary Diseases of Health Ministry, Tongji Hospital, Tongji Medical College, Huazhong University of Science and Technology, Wuhan, China

**Keywords:** necroptosis, ripk1, ripk3, mlkl, pulmonary diseases

## Abstract

In the past decades, apoptosis has been the most well-studied regulated cell death (RCD) that has essential functions in tissue homeostasis throughout life. However, a novel form of RCD called necroptosis, which requires receptor-interacting protein kinase-3 (RIPK3) and mixed-lineage kinase domain-like pseudokinase (MLKL), has recently been receiving increasing scientific attention. The phosphorylation of RIPK3 enables the recruitment and phosphorylation of MLKL, which oligomerizes and translocates to the plasma membranes, ultimately leading to plasma membrane rupture and cell death. Although apoptosis elicits no inflammatory responses, necroptosis triggers inflammation or causes an innate immune response to protect the body through the release of damage-associated molecular patterns (DAMPs). Increasing evidence now suggests that necroptosis is implicated in the pathogenesis of several human diseases such as systemic inflammation, respiratory diseases, cardiovascular diseases, neurodegenerative diseases, neurological diseases, and cancer. This review summarizes the emerging insights of necroptosis and its contribution toward the pathogenesis of lung diseases.

## Introduction

The normal growth and development of multicellular organisms depend on the balance between cell death, which clears damaged, infected, or redundant cells, and cell proliferation (Galluzzi et al., 2018). Two modalities of cell death have been classically recognized: one is accidental cell death caused by a sudden and intolerable physical, chemical, or mechanical factor, including necrosis, and the other is RCD via genetically encoded mechanisms, widely known as apoptosis (Degterev et al., 2005). RCD can occur in the absence of external stimuli, including organ development or tissue replacement, and is often referred to as programmed cell death. In addition, it can occur in cells that cannot adapt to the intracellular and extracellular microenvironments disturbed by stressors. Apoptosis is a prototypical form of RCD, characterized by cell membrane sclerosis, nuclear chromatin concentration, nuclear fragmentation, and plasma membrane bubbling. Necroptosis, termed so in 2005, is a regulated form of cell death that shares an upstream signaling pathway with apoptosis, and the underlying molecular mechanisms of the two processes considerably overlap (Linkermann and Green, 2014; Vanden Berghe et al., 2016). However, the death morphology of necroptosis is similar to necrosis, but not apoptosis, and is characterized by cell membrane hardening, nuclear chromatin concentration, nuclear fragmentation, and plasma membrane bubbling (Galluzzi et al., 2018).

Necroptosis also plays an important role in the early developmental stages (Lin et al., 2016; Shan et al., 2018), and is a defense mechanism against infectious diseases (Li et al., 2012; Wang X. et al., 2014). The key molecules of necroptosis are RIPK1, RIPK3, and MLKL. In tumor necrosis factor receptor (TNFR) 1-mediated necroptosis, RIPK1 combines with RIPK3 to form a necrotic complex, mediating the oligomerization of MLKL that is transported to the cell membrane, resulting in cell expansion and cell death (Shi et al., 2020). Early necroptosis was considered the alternative form of apoptosis; however increasing evidence has shown that necroptosis itself is associated with many clinical diseases. Numerous studies on diseases such as cardiovascular (Zhang et al., 2016; Li et al., 2019a; Sun T. et al., 2019), central nervous system (Yuan et al., 2019), digestive (Wang R. et al., 2020), infectious (Duprez et al., 2011; Wang R. et al., 2020), genetic diseases (Vitner et al., 2014; Morgan et al., 2018), and various tumors (Seifert et al., 2016; Strilic et al., 2016; Seehawer et al., 2018) have demonstrated that blocking the necroptotic pathway by drug inhibition, gene knockout, or knockdown shows satisfactory results and that promoting the occurrence of necroptosis in tumors can facilitate tumor inhibition (Zhao et al., 2015; Newton et al., 2016a).

Recently, necroptosis has been found to be involved in the occurrence and development of lung diseases such as lung infections, acute lung injury (ALI)/acute respiratory distress syndrome (ARDS), coronavirus disease 2019 (COVID-19), asthma, chronic obstructive pulmonary disease (COPD), idiopathic pulmonary fibrosis (IPF), pulmonary arterial hypertension (PAH), and lung cancer (Table 1). However, several questions remain to be addressed.

**TABLE 1:** The role of necroptosis in pulmonary diseases.

Disease	More specific	Main content about necroptosis	References
**In infection**	S. aureus pneumonia	The accessory gene regulator (agr) quorum sensing system can be inhibited by the heptapeptide RNAIII-inhibiting peptide, which dampens (Phenol-soluble modulins) PSMα-induced neutrophil necroptosis	Zhou et al. (2018)
		S. aureus activates the NLRC4 to suppress γδ T cell-derived IL-17A-dependent neutrophil recruitment by driving necroptosis and IL-18 production, which impedes host defense	Paudel et al. (2019)
		NLRP6 expression is increased, triggering necroptosis and hyper-inflammation via the TNF-α pathway, leading to the loss of neutrophils by dampening IFN-γ and ROS production	Ghimire et al. (2018)
		Agr-regulated toxins activate necroptosis and IL-1β expression, leading to alveolar macrophage depletion and lung injury	Kitur et al. (2015)
	Bacterial PFTs	PFTs-induced respiratory epithelial cell RIP1/RIP3/MLKL-dependent necroptosis, as a result of influenza-induced oxidative stress, was triggered by ion dysregulation through PFT-mediated membrane permeabilization	Gonzalez-Juarbe et al. (2017), Gonzalez-Juarbe et al. (2020)
		PFTs induce necroptosis of macrophages through ion dysregulation, mitochondrial damage, ATP depletion, and oxidative stress	Gonzalez-Juarbe et al. (2015)
		PETs-induced necroptosis plays a beneficial role in facilitating adaptive immune response through the release of inflammatory factors	Riegler et al. (2019)
	Klebsiella pneumoniae (KPn)	KPn infection damage the neutrophil efferocytosis by inducing necroptosis of neutrophils	Jondle et al. (2018)
	Mycobacterium tuberculosis (Mtb)	TNFα excess triggers ROS production then induces RIPK1-RIPK3-dependent necroptosis of macrophages, leading to bacterial dissemination	Roca and Ramakrishnan (2013)
		TNFα excess leads to RIPK1-RIPK3-dependent necroptosis in murine fibroblasts and RIPK1-dependent necrosis-like cell death in murine macrophages	Butler et al. (2017)
		Virulent Mtb evasion of macrophages apoptosis and immunity by Bcl-xL, inducing RIPK3–impendent necrosis and preventing caspase 8-activation	Zhao et al. (2017)
		The inhibition of necroptosis by MLKL-deficiency or Nec-1 in humanized mice does not affect Mtb infection progression	Stutz et al. (2018a)
		RIPK3 is not an important mediator of pathological inflammation or macrophage necrosis in Mtb, for the reason is that inhibition of RIPK3 is not effective	Stutz et al. (2018b)
	Influenza A virus	RIPK3 is activated by IAV and plays a crucial role in antiviral immunity by activating MLKL-dependent necroptosis with RIPK3 kinase activity and FADD-mediated apoptosis. ZBP1 is the link between IAV and RIPK3 activation, and ZBP1 deficiency is resistant to IAV-triggered necroptosis and apoptosis	Downey et al. (2017)
		RIPK3 is activated by IAV and plays a crucial role in antiviral immunity by activating MLKL-dependent necroptosis with RIPK3 kinase activity and FADD-mediated apoptosis. ZBP1 is the link between IAV and RIPK3 activation, and ZBP1 deficiency is resistant to IAV-triggered necroptosis and apoptosis	Nogusa et al. (2016), Thapa et al. (2016)
		Z-RNAs generated by replicating IAV activate ZBP1, activating RIPK3 and MLKL, thus leading to nuclear membrane rupture and resulting in a nucleus-to-cytoplasm necroptosis. In unrestrained cell death, MLKL-induced nuclear rupture causes exceeding and deleterious inflammatory responses, which drive IAV disease severity	Zhang et al. (2020)
	RSV	RSV induces RIPK3-MLKL-dependent necroptosis of macrophages by activating TLR4/TLR3 and pyroptosis through activating TLR2 and triggering ROS generation	Bedient et al. (2020)
**ALI/ARDS**	Influenza A H7N9 virus	Low expression of cIAP2 caused RIPK1/3-dependent necroptosis of airway epithelial cells, leading to ALI/ARDS and death	Qin et al. (2019)
	OA-induced ALI/ARDS	RIPK3/MLKL-independent necroptosis is obviously activated, while lung edema and inflammation is reduced by Nec-1	Pan et al. (2016b)
	LPS-induced ALI/ARDS	Plasma RIPK3 is associated with ALI/ARDS. RIPK3 depletion reduced inflammatory mediators and ameliorated lung tissue injury	Wang et al. (2016), Shashaty et al. (2019)
		LPS induces ZBP-1 expression, which causes RIK3/MLKL-dependent necroptosis that results in the release of DAMPs to activate the TLR9/NF-κB pathway and macrophages release pro-inflammatory cytokines, leading to lung inflammation and injury	Du et al. (2019)
		The expression of CXCR1/2 and p-MLKL is high, and the SP level is high while the VIP level is low. All could be reversed by reparixin, a CXCR antagonist that increased the survival rate mice of mice and improved lung inflammation	Wang et al. (2018b)
	hyperoxic acute lung injury (HALI)	Hyperoxia exposure causes necroptosis with increased expression of RIPK1, RIPK3, and MLKL by oxidative stress, leading to inflammatory infiltration and pulmonary edema. Hyperoxia-induced miR-185-5p promotes both apoptosis and necroptosis	Han et al. (2018), Carnino et al. (2020)
	Hydrogen sulfide (H2S)	H2S exposure results in lung injury, immune suppression, inflammatory response, and necroptosis or other cell death. LncRNA3037/miR-15a/BCL2-A20 signaling could be involved in these	Li et al. (2020b), Liu et al. (2020b)
	Ventilator-induced lung injury (VILI)	Plasma RIPK3 levels are higher in patients with mechanical ventilation (MV) and RIPK3 deficiency confer protection against VILI.	Siempos et al. (2018)
	Red blood cell (RBC) transfusions	RBC transfusion triggers RIPK3-dependent necroptosis of lung endothelial cells with the release of HMGB1, leading to lung inflammation and damage. Advanced Glycation End Products (RAGE) could be an essential mediator	Qing et al. (2014), Faust et al. (2020)
	Lung transplantation	Prolonged cold-ischemia-induced ischemia-reperfusion causes RIPK3/MLKL-dependent necroptosis via calpain-STAT3-RIPK axis activation, leading to predisposing lung grafts to primary graft dysfunction (PGD)	Kim et al. (2018), Wang et al. (2019a)
	Renal allografts	Regulated necrosis including parthanatos and necroptosis involve in part of the mechanism of renal graft injury that leads to lung injury, and necroptosis mediated by OPN signaling in pancreatitis-associated lung injury	Zhao et al. (2015), Zhao et al. (2019a)
**SARS-CoV-2**		SARS-CoV-2 activates caspase-8, leading to caspase-8-mediated apoptosis and inflammatory response, and RIPK3-MLKL-dependent necroptosis without fully inhibited by caspase-8. The dual modes of cell death pathways play a dual role in appropriately immune responses to restrict viral replication or severe lung damage as a hyperactivation status	Li et al. (2020a)
		The combination of TNF-a and IFN-g induced the JAK/STAT1/IRF1 axis activation, leading necroptosis and other inflammatory cell death processes that could be one of the possible mechanisms linking cytokine storm to organ damage	Karki et al. (2021)
**Asthma**	RSV	RSV-induced necroptosis results in the release of HMGB1 and neutrophilic that contributes to RSV bronchiolitis pathogenesis inflammation. Inhibition of necroptosis attenuated the pathologies that will ameliorate asthma progression in later-life	Simpson et al. (2020)
	MUC1	TNF-α could induce necroptosis of 16HBE cells accompanied by the upregulation of MUC1, while MUC1 downregulation increase necroptosis and inhibit the effects of anti-necroptosis by Dex	Zhang et al. (2019a), Zhang et al. (2019b)
	Aspergillus-induced asthma model	RIPK3-MLKL necroptosis induce the release of bioactive IL-33, which can activate basophils and eosinophils, leading to exacerbating of allergic inflammation	Shlomovitz et al. (2019)
	Adhesion-induced eosinophil cytolysis	RIPK1-independent necroptosis take part in adhesion-induced eosinophil cytolysis, which is required p38 MAPK and NADPH oxidase activation	Radonjic-Hoesli et al. (2017)
	particulate matter (PM)	PM2. 5 results in airway Hyperresponsiveness and trachea injury by necroptosis, which induces neutrophils and IL-17 to inflammation	Zhao et al. (2019b)
**COPD**	PM	Airborne PM exposure induces oxidative stress that can trigger necroptosis, leading to PM-induced pulmonary inflammation and mucus hyperproduction	Peixoto et al. (2017), Xu et al. (2018)
	Cigarette Smoke (CS)	Induces necroptosis of lung structural cells with a release of DAMPs, leading to neutrophilic airway inflammation, which is suppressed with inhibition of GRP78	Pouwels et al. (2016), Wang et al. (2018c), Wang et al. (2020c)
		Triggers mitophagy-dependent necroptosis via PINK1 stabilization with mitophagy, in which C16-Cer could be an upstream initiator, while highC24-DHC levels might protect against CS-induced necroptosis	Mizumura et al. (2014), Mizumura et al. (2018)
**Idiopathic Pulmonary Fibrosis**	BLM-induced model	The level of RIPK3 expression is increased in lung tissue from IPF patients. ROS production by BLM triggers RIPK3-dependent necroptosis, which takes part in fibrosis development through inflammatory cell accumulation via the release of DAMPs	Lee et al. (2018)
	SFTPA1	JNK-mediated the overexpress of RIPK3, which triggers necroptosis of AEII cells in Sftpa1-KI mice, leading to pulmonary fibrosis	Takezaki et al. (2019)
**Pulmonary arterial hypertension**	PAH severity	Necroptosis and necrosis play a potential role in HMGB1 release, activation of TLR4, and the manifestation of sex difference in PAH severity	Zemskova et al. (2020)
	Monocrotaline-induced PAH	RIPK3-mediated necroptosis is involved in the generation of DAMPs that was associated with the activation of TLR and NLR pathways by bioinformatics analysis	Xiao et al. (2020)
**Lung cancer**	Metastasis	Induce necroptosis of endothelial cells leading to extravasation and metastasis via amyloid precursor protein and DR6, a primary mediator	Strilic et al. (2016)
		TAK1 deficiency is more likely to cause RIPK3-dependent necroptosis of human/murine endothelial cells by Up-regulating the expression of RIPK3 and form metastases by endothelial	Yang et al. (2019)
	Prognosis in NSCLC	The high level of RIPK3 was associated with improved local control(LC) and progression-free survival (PFS) after hypofractionated radiation therapy. But low RIPK3 showed worse disease free survival (DFS) after curative resection and worse chemotherapy response	Wang et al. (2018a), Park et al. (2020), Wang et al. (2020a)
		Higher RIPK3 expression is associated with a shorter OS and a tendency of shorter DFS, which reason might be the effect of resistance to radiotherapy or excessive necroptosis-mediated damage	Kim et al. (2020)

## The Mechanism of Necroptosis and Its Relationship With Apoptosis

**FIGURE 1:**
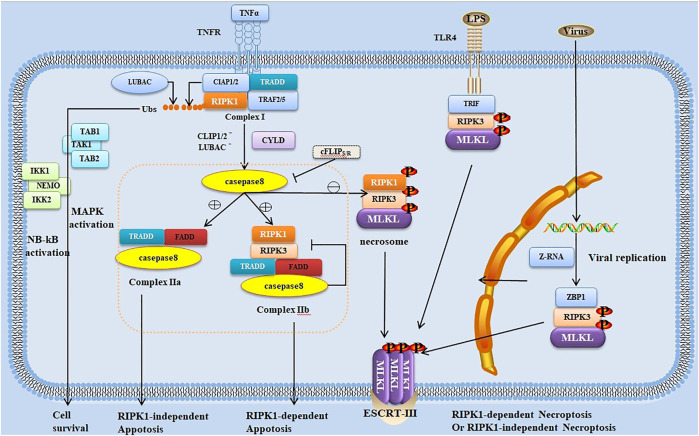
Trigger and regulation of signal transduction pathways in necroptosis and apoptosis. Necroptosis typically occurs as a consequence of the stimulation of tumor necrosis factor receptor (TNFR) 1 by TNF, leading to the formation of receptor-bound complex I such as TNFR1-associated DD (TRADD), receptor-interacting protein (RIP) kinase (RIPK) 1, TNF-associated factor 2/5 (TRAF2/TRAF5), cellular inhibitor of apoptosis proteins (cIAPs), and linear ubiquitin chain assembly complex (LUBAC). RIPK1 ubiquitination by LUBAC or cIAPs results in the recruitment of I-κB kinase (IKK) complex (including IKK1, IKK2, and the nuclear factor-kappa B (NF-κB) essential modulator [NEMO]) and transforming growth factor-β-activated kinase 1 (TAK1)-binding protein (TAB) complex, leading to the activation of mitogen-activated protein kinase and NF-κB for cell survival. Cylindromatosis (CYLD) and A20 deubiquitinate RIPK1, which is released from complex I, leading to the transition from complex I to cytosolic complex II. The binding of TRADD–Fas-associated death domain protein (FADD)–caspase-8, one complex called complex IIa, triggers RIPK1-independent apoptosis, whereas the other complex IIb (comprising RIPK1, RIPK3, FADD, and caspase-8) triggers RIPK1-dependent apoptosis. Moreover, RIPK1 can inhibit FADD–caspase-8-dependent apoptosis. Caspase-8 can be inhibited by c-FLIP as a result of heterodimerization between c-FLIPS and procaspase-8. With the reduction, blockage, or absence of cIAPs or caspase-8, the necrosome (comprising RIPK1, RIPK3, and MLKL) is formed, which is the binding of activated RIPK1 and RIPK3, leading to the activation and phosphorylation of RIPK3. RIPK3 then activates MLKL, which is transferred to the cell membrane, ultimately leading to cell expansion and rupture and RIPK1-dependent necroptosis. RIPK3 can be activated not with RIPK1 but with other RIP homotypic interaction motif (RHIM)-containing proteins such as TLRs. TLRs can induce RIPK3 activation and RIPK1-independent necroptosis through the RHIM-containing adapter TIR domain-containing adaptor-inducing interferon-β (TRIF). In the nucleus of influenza virus-infected cells, Z-RNAs are produced when replicated viruses are sensed by the host Z-DNA-binding protein 1 (ZBP1), activating RIPK3 independent of RIPK1 by its RHIM domain.

The necroptosis pathway is initiated by activating Z-nucleic acid binding protein 1 (ZBP1, also known as DAI or DLM-1) (Zhang et al., 2020) or numerous ligand-dependent receptors, including tumor necrosis factor receptor (TNFR), interferon receptors (IFNRs) and Toll-like receptors (TLRs) (Choi et al., 2019). Even the necroptosis pathway can be independent of the death receptor signal when ion imbalance due to pore-forming toxin (PFT)-mediated membrane permeability is activated (Hakansson and Bergenfelz, 2017). The most typical one is the signaling pathway induced by TNFR1 (Vanden Berghe et al., 2014; Fuchs and Steller, 2015). Both RIPK1 and RIPK3 have RHIM at the C-terminal. TNFR1 activation leads to the recruitment of RIPK1 and other adapter proteins (Figure 1). Subsequently, RIPK1 and RIPK3 bind through RHIM to form an amyloid signaling complex called the necrosome (Pasparakis and Vandenabeele, 2015). RIPK3, which has a phosphorylation on Ser232 in the necrosome, recruits and phosphorylates the executioner MLKL. The phosphorylated MLKL undergoes conformational changes and disengagements from RIPK3; then, the MLKL oligomer is transferred to the cell membrane, leading to cell expansion and rupture (Wang H. et al., 2014; Garnish et al., 2021). Recently, it has been found that MLKL ubiquitination is also correlated with MLKL activation and necroptosis, and MLKL ubiquitination at K219 imposes the cytotoxic potential of phosphorylated MLKL (Garcia et al., 2021). Cell rupture is followed by the release of a large number of endogenous host-derived molecules such as adenosine triphosphate, IL-33, heat-shock proteins, and high-mobility group box 1 (HMGB1) and other DAMPs, thus causing an excessive inflammatory response and aggravating surrounding tissue damage (Scaffidi et al., 2002; Iyer et al., 2009; Gong et al., 2020).

As a typical regulatory cell death program, apoptosis is primarily mediated by caspase-mediated exogenous and endogenous signaling pathways. Death receptors, including Fas/FasL and TNF-1/TNF-α, are typical molecular models in exogenous signals that lead to cell apoptosis. Binding of these receptors and ligands to the caspase-8/caspase-10 precursor protein through the intracellular adaptor protein Fas-associated DD protein (FADD) activates procaspase and thus initiates the caspase cascade. Then, further activation of downstream effector molecules leads to apoptosis. Caspase is the key to many apoptotic regulatory pathways (Choi et al., 2019; Schwarzer et al., 2020). Upon the ligand activation of TNFR1, RIPK1 is ubiquitinated by the cellular inhibitors of apoptosis proteins (cIAPs) and/or linear ubiquitin chain assembly complex (LUBAC), which subsequently promotes inflammation or cell survival. cIAPs or LUBAC defects result in RIPK1 ubiquitination failure, ultimately leading to apoptosis or necroptosis (Dondelinger et al., 2015; Geng et al., 2017; Wei et al., 2017). The subsequent outcomes depend on caspase-8, which can mediate the hydrolysis and lysis of RIPK1 and RIPK3. It is like a switch, and its availability is involved in determining whether necroptosis or apoptosis would occur. With the presence of caspase-8, deubiquitination of RIPK1 mediates cell apoptosis, whereas the absence of caspase-8 results in the failure of RIPK1/RIPK3 proteolytic effect, thus leading to cell necroptosis (Pasparakis and Vandenabeele, 2015) (Figure 1). However, macrophages infected with murine cytomegalovirus with M36 (a viral inhibitor of caspase activation)/M45 (a viral inhibitor of RIP activation) double mutation have been reported to also undergo secondary necroptosis even if caspase-8 is fully activated; this may have some unclear links with apoptosis (Daley-Bauer et al., 2017).

The relationship between necroptosis and apoptosis is complex, and the network of their molecular mechanisms is intricate (Cook et al., 2014; Newton et al., 2016a). In a mouse model of systemic inflammatory response syndrome (SIRS), the combined loss of MLKL (or RIPK3) and caspase-8 provides significant protection, suggesting that necroptosis and apoptosis coexist in the inflammation mechanism (Newton et al., 2016a). Both apoptosis and necroptosis have also been found in influenza A and severe acute respiratory syndrome coronavirus 2 (SARS-CoV-2), and the appropriate activation of both is believed to be effective in limiting the virus, whereas excessive activation can cause severe lung injury and thus fatal infection (Li S. et al., 2020; Gonzalez-Juarbe et al., 2020).

Actually, various cell deaths likely occur simultaneously, and recent studies have discovered the coregulation and crosstalk among these seemingly different cell death complexes. Pyroptosis is an well-known inflammatory form of cell death that requires the membrane damaging gasdermin D (GSDMD) cleaved and activated by caspase-1/4/5/11. Additionally, necroptosis and pyroptosis have some overlapping downstream signals, such as trigger the NLRP3 inflammasome to result in IL-1β-driven inflammation (Frank and Vince, 2019). A new concept called PANoptosis (pyroptosis, apoptosis, and necroptosis), which is a collective inflammatory cell death pathway and engaged by a multiprotein complex called the PANoptosome, has emerged (Samir et al., 2020; Zheng and Kanneganti, 2020). A study found that TNF-α and IFN-γ synergism mediated RIPK1/FADD/CASP8 axis-derived PANoptosis in murine bone marrow-derived macrophages (BMDMs) drives pathology in COVID-19 and other diseases associated with cytokine storm (Karki et al., 2021). But it is not clear why pyroptosis, apoptosis and necroptosis are tightly connected and crossregulated each other. It is reported that Caspase-8 could be the molecular switch that controls PANoptosis to prevent tissue damage during embryonic development and adulthood (Fritsch et al., 2019). Given the complexity of cell deaths, it is necessary to exclude the interference of other cell deaths, by treating with the inhibitors or specific knockout of key molecules in one cell death, when explore the others. For example, some studies exploring the mechanism of necroptosis will block apoptosis by treatment with zVAD-fmk, in order to clarify the role of necroptosis the pathogenesis of diseases more intently (Li et al., 2019b; Kishino et al., 2019). However, there are few studies on similar combinations among other cell deaths.

## Complex Network Among Related Molecules of Necroptosis

RIPK1, RIPK3, and MLKL are important in necroptosis signaling pathway. The mutual restriction of death molecules plays a crucial role in regulating the balance between cell death and survival, although adverse consequences can easily occur. RIPK1 is not always involved in necroptosis (Upton et al., 2019), but it may serve as a regulator with distinct mechanisms for different modes of regulation in cell death. As a kinase, the kinase activity of RIPK1 induces RIPK3 activation and necroptosis (Geng et al., 2017). Recently, growing evidences have shown that RIPK1 phosphorylation at different sites by different kinases may regulate the kinase activity (Degterev et al., 2008; Laurien et al., 2020). Autophosphorylation of RIPK1 regulates the kinase activity of RIPK1, which may aid in particular conformational changes to facilitate necroptosis and apoptosis (Wegner et al., 2017). RIPK1 autophosphorylation at S166 regulates the kinase activity, but it cannot impose conformational changes on RIPK1 to activate the downstream cell death signaling (Laurien et al., 2020). However, phosphorylation of RIPK1 by kinases, including MAPK-activated protein kinase 2 (MK2) that phosphorylates RIPK1 at Ser321/336, TAK1 that phosphorylates RIPK1 at Ser321, NF-κB kinases (IKKs) that phosphorylate RIPK1 on multiple residues, TANK binding kinase 1 (TBK1), and IkappaB kinase epsilon (IKKε), results in the inhibition of RIPK1 kinase activity and prevents TNF-mediated RIPK1-dependent cell death (Dondelinger et al., 2015; Koppe et al., 2016; Geng et al., 2017; Jaco et al., 2017; Menon et al., 2017; Lafont et al., 2018). Recent studies have suggested that ZBP1 promotes caspase-8-mediated cell death and inflammasome activation by RHIM-mediated interactions with RIPK1, which depends on the kinase activity of RIPK1 (Muendlein et al., 2021).

The kinase-independent and scaffold-like functions of RIPK1, which mediates NF-κB and MAPK activation, are essential for cell survival. In various disease models, blocking the necroptosis pathway plays a role in promoting survival. However, interestingly, RIPK1 can inhibit necroptosis and apoptosis in the early embryonic stage during development, playing a protective role during development (Dillon et al., 2014; Newton et al., 2016b; Anderton et al., 2019; Jiao et al., 2020). In some mouse models of development, RIPK1 inhibits FADD–caspase-8-dependent apoptosis by blocking FADD recruitment by TNFR1-associated DD and preventing abnormal caspase-8 activation. Similarly, RIPK3-dependent necroptosis driven by ZBP1 can be inhibited by RIPK1, which can prevent the binding of ZBP1 containing RHIM to RIPK3 (Newton et al., 2016b). On the basis of previous studies, mice that express RIPK1 (D325A), with a mutation in the caspase-8 cleavage site Asp325, died mid-gestation; this effect is consistent with that of a lack of caspase-8 (Newton et al., 2019; Lalaoui et al., 2020). However, evidence shows that RIPK1 can be a central driver of inflammation through its scaffold function to activate the NF-κB pathway and promote inflammatory cytokine release in the early phase; the inflammatory processes is RIPK1 kinase–dependent in the late phase (Huang et al., 2021).

In contrast to RIPK1, RIPK3 is indispensable in necroptosis (He et al., 2009; Upton et al., 2019). Various molecules can bind to RIPK3 with RHIM (Dillon et al., 2014), including ZBP1 and TIR domain-containing adaptor-inducing interferon-β (TRIF), leading to RIPK1-independent necroptosis (He et al., 2011; Kaiser et al., 2013; Thapa et al., 2016; Upton et al., 2019). RIPK3 can also mediate necroptosis and apoptosis (Cook et al., 2014; Mandal et al., 2014; Newton et al., 2014). Interestingly, RIPK3 also plays an important role in being independent of necroptosis. Studies have found that in lipopolysaccharide (LPS)-induced mouse models, RIPK3 triggers the activation of nucleotide-binding domain (NOD)-like receptor family pyrin domain-containing 3 (NLRP3) inflammasomes independent of necroptosis, which indirectly illustrates the richness of RIPK3 (Lawlor et al., 2015; Chen et al., 2018; He and Wang, 2018). In mouse models of West Nile virus (WNV) encephalitis, RIPK3 can enhance the expression of neuronal chemokines and coordinate the immune response in the central nervous system. The Ripk3^−/−^mice showed higher mortality with defects in recruiting immune cells than the wildtype mice, while neither Mlkl^−/−^ nor Mlkl^−/−^Casp8^−/−^mice exhibited the defects. These data identify that RIPK3 plays a role in inhibiting the pathogenesis of WNV independent of necroptosis (Daniels et al., 2017).

MLKL is associated with endosomes and assists in endosome transport and extracellular vesicle production, independent of necroptosis (Yoon et al., 2017). Cells harboring active MLKL may not be inevitably lethal. The endosomal sorting complex required for transport-III can temporarily protect cells undergoing necroptosis to express functional proteins (Gong et al., 2017). Aside from being the executioner of necroptosis, MLKL can indirectly regulate gene expression, block specific processes via affinity for selected molecules (Zhan et al., 2021), affect cancer development and metastasis (Martens et al., 2021), and promote inflammation by activating NLRP3 (Conos et al., 2017).

## The Role of Necroptosis in Lung Diseases

### Pathogen Infection

Necroptosis has a certain defensive effect in the fight against some microorganisms or toxins infection, but it can also cause excessive inflammation and aggravate tissue damage by releasing DAMPs. Whether necroptosis plays a protective or injurious role in infectious disease may depend on the kinds of pathogen, the cell type and the degree of inflammation.

In bacterial pneumonia, most studies mention that necroptosis is detrimental. The necroptosis of immune cells, including macrophages or neutrophils, leads to the expansion of inflammatory response and increases the damage of the body; it can be alleviated by inhibiting necroptosis in mouse bacterial pneumonia model. Bacterial pathogens can cause the necroptosis of lung epithelial cells through PFTs, consumption of macrophages, or non-death receptor-dependent necroptosis through ion imbalance in the mouse pneumonia model (Gonzalez-Juarbe et al., 2015; Gonzalez-Juarbe et al., 2017; Hakansson and Bergenfelz, 2017; Gonzalez-Juarbe et al., 2018). However, PFT-induced necroptosis plays a beneficial role in facilitating adaptive immune response through the release of inflammatory factors (Riegler et al., 2019). Virulent pneumococcal strains with low NF-κB activation potential were shown to induce macrophage necroptosis, resulting in higher bacterial burden and pneumonia severity (Hakansson and Bergenfelz, 2017). In Staphylococcus aureus (S. aureus) pneumonia, immune cells such as including neutrophils and macrophages are the key to immune defense, although S. aureus can inhibit host defense by inducing the necroptosis of neutrophils and lung macrophages, thereby aggravating tissue damage and even causing the occurrence of ARDS; however, it can enhance the clearance of S. aureus and reduce lung damage by inhibiting the occurrence of necroptosis (Kitur et al., 2015; Ghimire et al., 2018; Zhou et al., 2018; Du et al., 2019; Paudel et al., 2019). A regulated process against infection called neutrophil efferocytosis impairment by Klebsiella pneumoniae (KPn) through the activation of necroptosis machinery is restored with RIPK-1 inhibitor Necrostatin (Nec)-1s or RIPK3 inhibitor GSK’872, which improve the overall disease outcome in KPn-infected mice (Jondle et al., 2018).

Interestingly, necroptosis has different effects in Mycobacterium tuberculosis (Mtb) infection. In zebrafish, the excess of TNF induces Mtb-infected macrophage necroptosis through mitochondrial reactive oxygen species (ROS) production, which releases mycobacteria into the growth-permissive extracellular milieu and disseminate *Mycobacterium* lentiflavum infection (Roca and Ramakrishnan, 2013). In contrast, necroptosis inhibition by MLKL-deficiency or Nec-1 in humanized mice does not affect Mtb infection progression because macrophage necroptosis is ultimately restricted to mitigate disease pathogenesis (Stutz et al., 2018a). Similarly, RIPK3 does not play a fundamental role in regulating inflammatory responses or necrotic macrophage death *in vivo*. Because compared with wild-type mice, RIPK3-deficient mice do not indicate the benefit of reducing the bacterial burden (Stutz et al., 2018b).

In viral pneumonia, necroptosis could be a protection against and a way for infected lung cells to restrict viral replication (Pasparakis and Vandenabeele, 2015). RIPK3-dependent necroptosis is required for protection against Vaccinia virus (VV) infection in VV-infected mice, and RIPK3−/−mice exhibit severely impaired tissues, inflammation, and uncontrollable virus replication (Cho et al., 2009). RIPK3 plays an important role in sequestering viral replication and protecting the mice against influenza A virus (IAV) infection by regulating type I IFN signaling at both the transcriptional and posttranscriptional levels. Moreover, ZBP1, a sensor of RNA viruses, is the link between IAV replication and RIPK3 activation. ZBP1-/RIPK3-deficient mice are hypersusceptible to lethal infection caused by IAV, failing to control IAV replication and succumbing to lethal respiratory infection (Nogusa et al., 2016; Thapa et al., 2016; Downey et al., 2017; Wang Y. et al., 2019). Emerging evidence shows that Z-RNAs have been generated by replicating IAV-activated ZBP1, activating MLKL in the nucleus of infected cells, and lead to nuclear membranes rupture, resulting in an “inside-out” (i.e., nucleus-to-cytoplasm) cell death. When cell death is unrestrained, MLKL-activated nuclear envelope rupture releases nuclear DAMPs, promoting the recruitment and activation of neutrophils, which then contribute to serious consequences in mice (Zhang et al., 2020). MLKL-deficient mice had reduced IAV disease severity during secondary bacterial infection (Gonzalez-Juarbe et al., 2020). Collectively, necroptosis is an effective mechanism for the clearance of some viruses from host cells. However, SARS-CoV-2 infection triggers uncontrollable apoptosis and necroptosis, leading to lung damage in SARS-CoV-2-infected hepatocyte nuclear factor-3/forkhead homolog 4-(HFH4-) human angiotensin-converting enzyme 2 (hACE2) transgenic mouse model (Li S. et al., 2020). Similarly, respiratory syncytial virus (RSV) induces lytic cell death in the human monocyte cell line (THP-1) via RIPK3-MLKL mediated necroptosis and apoptosis-associated speck-like protein containing a caspase recruitment domain-(ASC-) NLRP3 inflammasome-dependent pyroptosis. The combined treatment of GSK’872 (the RIPK3 specific inhibitor) and zVAD-fmk (the pan-caspase inhibitor) exhibits less of lytic cell deaths than a single treatment (Bedient et al., 2020). When cell death is unrestrained, injury and severe illness follow. Although accumulating evidence demonstrates that necroptosis has a defensive effect in the fight against some microorganisms or toxins infection, further investigation is needed to elucidate the importance of necroptosis in the pathogenesis of infection diseases. And the mechanism that triggers cell death to fight infection without damaging the body needs to be explored.

### ALI/ARDS

Several predisposing factors for ALI/ARDS exist, which include infection, trauma, and systemic inflammation. As mentioned above, lung infections, including S. aureus infection and influenza, and other systemic inflammation can result in severe lung damage, even ARDS. Much evidence shows that necroptosis is an important mechanism of inflammation that leads to lung injury. Some microorganisms or proinflammatory mediators mediate the necroptosis of immune cells, including macrophages and lung epithelial cells with significantly elevated RIPK3/MLKL. Then MLKL-induced membrane ruptures with the release of DAMPs, causing an excessive inflammatory response and aggravating lung tissue damage in mice (Wang et al., 2016; Chen et al., 2018; Fan and Fan, 2018). Both inflammation and the degree of lung injury are reduced with Nec-1, which may attenuate oxidative stress (Wang et al., 2016; Lin et al., 2020). In a neonatal septic mouse induced by intraperitoneal injection of adult cecal slurry, RIPK1 inhibition by Nec-1 has been reported to play a protective role, decreasing lung injury and increasing survival (Bolognese et al., 2018).

Growing evidence has gradually revealed the regulatory factors of necroptosis in ARDS. The death of pulmonary epithelial and infected cells is the cause of death in ALI/ARDS with A/H7N9 virus infection. cIAPs play an important role in cell survival by regulating the necroptosis pathway. However, cIAP2 is significantly downregulated and RIPK3 is increased in the lung tissues of patients who die from H7N9 infection. Collectively, a study found that necroptosis is associated with severe H7N9 infection in lung tissues from patients who died from ARDS-complicated H7N9 infection, leading to ARDS and even death, which can be regulated by cIAP2 (Qin et al., 2019). HSP90 is found to be required for RIPK3 activation through the modulation of the stability of MLKL and promotion of MLKL oligomerization and plasma membrane transformation in a mouse model of severe ARDS (Yu et al., 2020).

Some noninfectious contributing factors can induce ARDS or aggravate lung damage in ARDS. RBC transfusion sensitizes mice to LPS-induced lung inflammation through the release of the danger signal HMGB1 and induces lung endothelial cell necroptosis, which sensitizes the lung to subsequent injury (Qing et al., 2014). Some studies have demonstrated that fatty acid oxidation-dependent RIPK3 mediates the pathogenesis of ALI in patients requiring ventilator support, whereas RIPK3^−/−^ mice sustained less severe ventilator-induced lung injury than wild-type mice (Siempos et al., 2018). In rats exposed to pure oxygen to induce hyperoxic ALI, induced oxidative stress may activate necroptosis that causes lung damage, whereas necroptosis inhibition can improve lung pathology (Han et al., 2018). In ATII cells, hyperoxia and its derivative, ROS, upregulate miR-185-5p, which can regulate both necroptosis and apoptosis by suppressing FADD and caspase-8 (Carnino et al., 2020). In a pig lung model exposed to hydrogen sulfide (H2S), necroptosis is associated with H2S-induced lung injury (Liu Z. et al., 2020). Necroptosis is significantly activated in ischemia-reperfusion injury after prolonged cold ischemic time in lung transplantation of rats/mice, which may be a key event contributing to primary graft dysfunction, whereas Nec-1 leads to a significant decrease in pathologic epithelial injury (Kanou et al., 2018; Wang X. et al., 2019). Necroptosis apparently mediated through osteopontin signaling has been reported to be associated with renal allograft transplant that triggers recipient remote lung injury in rats (Zhao et al., 2015; Zhao H. et al., 2019). Similarly, in a rat model of ARDS induced by oleic acid, necroptosis was significantly activated (Pan et al., 2016a; Pan et al., 2016b). Accordingly, necroptosis contribute to excessive inflammatory damage; manipulating necroptosis could provide new therapeutic opportunities in reducing lung damage and the severity of ARDS/ALI.

### COVID-19

COVID-19 is caused by the novel SARS-CoV-2, with its clinical manifestations ranging from no symptoms to ARDS and even death. Severe COVID-19 is characterized by the excessive production of proinflammatory cytokines and hypercoagulability as the result of an imbalance between the innate immune system and coagulation (Jose and Manuel, 2020; Moore and June, 2020; Karki et al., 2021). SARS-CoV-2 infection results in immune cell activation. This immune response can activate coagulation pathways, leading to proinflammatory cytokine overproduction, which is described as the COVID-19 cytokine storm, and multiorgan injury (Jose and Manuel, 2020). The processes of cell death, such as pyroptosis, apoptosis, and necroptosis, may be the mechanisms that link COVID-19 cytokine storm to organ damage (Karki et al., 2021). These processes require the specific combination of TNF-α and IFN-γ with signal transducers and activators of transcription 1 (STATs)/IFN-regulatory factor 1 (IRF-1) axis, which regulates the inducible nitric oxide synthase expression for NO production. Cells stimulated with TNF-α and IFN-γ show MLKL and RIPK1 phosphorylation, suggesting that necroptosis is implicated in the COVID-19 cytokine storm. The deletion of both RIPK3 and caspase-8 is found to protect against cell death, not RIPK3 deficiency only, which shows that not only necroptosis but also other processes of cell death occur in COVID-19 (Karki et al., 2021). A previous study has reported that SARS-CoV-2-induced secretion of inflammatory cytokines, including IL-1β, depend on caspase-8 activation that triggers cell apoptosis and necroptosis pathways in the lung sections of a SARS-CoV-2-infected HFH4-hACE2 transgenic mouse model. In addition, the phosphorylation of MLKL was upregulated in the plasma of Calu-3 cells with SARS-CoV-2 infection, whereas that of pMLKL was inhibited with the inhibition of RIPK3. This suggests that SARS-CoV-2 infection trigger the apoptosis and necroptosis pathways (Li S. et al., 2020). A recent study has reported that platelets incubated with infectious viruses appeared to undergo necroptosis and apoptosis. Phospho-MLKL and caspase-3 are increased in platelets of patients with COVID-19, showing that the necroptosis and apoptosis of platelets mediate a rapid response to SARS-CoV-2 (Koupenova et al., 2021).

Moreover, RIPK1 is a key protein in tissue-specific networks; it extensively interacts with other proteins, which suggests that RIPK1 plays an important role in inflammation or tissues damage in SARS-CoV-2 infection (Feng et al., 2020). RIPK1 activation is detected in the respiratory tract epithelium of patients with SARS-CoV-2 infection, whereas no phospho-RIPK1-positive cells are noted in the healthy individuals (Feng et al., 2020). Similarly, previous studies have demonstrated that the serum levels of RIPK3 were higher in 10 patients with COVID-19 and ARDS than in six with mild diseases, suggesting that RIPK3-mediated signal is associated with ARDS development in patients with COVID-19 (Nakamura et al., 2020). Collectively, necroptosis, the form of cell death that can trigger inflammatory responses by releasing inflammatory cytokines, may play a part in the pathogenesis and severity of COVID-19. Given that pyroptosis, apoptosis, and necroptosis are tightly connected and can crossregulate each other in COVID-19, further studies are warranted to explore the mechanism of cell death in COVID-19 to facilitate the development of therapeutic strategies.

### Asthma

Asthma, which is characterized by airway remodeling, airway hyperresponsiveness (AHR), and reversible airway obstruction, is a heterogeneous chronic inflammatory respiratory disease induced by eosinophils (Okano et al., 2015)_._ The inflammatory response after the release of granule by either cytolysis or degranulation of eosinophils causes increased damage in the airway epithelium and drives airway remodeling, which has been associated with asthma severity (Wark and Gibson, 2003; Okano et al., 2015; Eng and DeFelice, 2016). RIPK1-independent necroptosis has been reported to be the most likely pathway leading to eosinophil cytolysis, which can be counterregulated by autophagy (Radonjic-Hoesli et al., 2017). In a mouse model of allergic inflammation, Aspergillus fumigatus extract-induced asthma *in vivo* shows that bioactive IL-33, a proinflammatory cytokine, released during tissue damage to activate basophils and eosinophils is directly induced by necroptosis, which is blocked by GW806742X, a murine MLKL inhibitor (Shlomovitz et al., 2019). TNF-α is a well-known important cytokine in patients with asthma, which plays a central role in the development of AHR and other features of asthma (Brightling et al., 2008). TNF-α induces necroptosis of human bronchial epithelial (16HBE) cells accompanied by the upregulation of mucin 1 (MUC1), a membrane-tethered mucin glycoprotein, whereas MUC1 downregulation increases TNF-α-induced 16HBE cell necroptosis (Zhang et al., 2019a). Similarly, dexamethasone (Dex) has antinecroptosis effects on 16HBE cell, which is inhibited by the downregulation of MUC1 with the inhibition of glucocorticoid receptor-α nuclear translocation and attenuates the inhibitory effect of Dex on phosphorylated p65 (Zhang et al., 2019b). MUC1 may serve a protective role in antinecroptosis effects, which should be a potential target for the development of novel therapeutics for asthma.

In a mouse model of asthma exacerbations induced by in-house dust mite for inflammation and double-stranded RNA for exacerbation, both cell death markers, MLKL phosphorylation and lactate dehydrogenase, were increased and were observed more in IFNβ^−/−^ mice; thus, IFN-β deficiency may be a regulator of necrosis and necroptosis (Cerps et al., 2018).

RSV infection fails to induce apoptosis, but necroptosis, leading to HMGB1 release and neutrophilic inflammation that both contribute to RSV bronchiolitis pathogenesis in RSV-infected hAECs and murine pneumovirus infected mice. Treatment with Nec-1s/GW806742X in murine pneumovirus infected mice attenuates the pathologies by decreasing viral load and preventing type-2 inflammation and airway remodeling, which will ameliorate asthma progression in later life (Simpson et al., 2020). Fine particulate matter (PM) having a diameter <2.5 µm is a well-recognized risk factor for asthma. PM2.5 can enhance AHR and trachea injury by necroptosis in BALB/c mice, inducing neutrophils and IL-17 to cause inflammation (Zhao Y. et al., 2019). Overall, necroptosis has been implicated in the severity and pathological features of asthma and necroptosis inhibition may have beneficial effects on asthma.

### COPD

COPD, a heterogeneous and complex disease, is a progressive inflammatory disease of the airways, alveoli, and microvasculature. Cigarette smoking (CS) and indoor air pollution are common risk factors for developing COPD (Rabe and Watz, 2017; Roy, 2019). Increasing evidence suggests that multiple forms of cell death such as apoptosis, necrosis, necroptosis, and autophagy have been widely implicated in COPD pathogenesis (Kaup et al., 1990; Yokohori et al., 2004; Ryter et al., 2009; Pouwels et al., 2016). CS exposure induces necroptosis in lung structural cells with the release of DAMPs, leading to neutrophilic airway inflammation in mice (Pouwels et al., 2016; Wang Y. et al., 2018). However, CS-induced necroptosis is significantly suppressed with the inhibition of GRP78, a member of the HSP70 family (Wang Y. et al., 2018). Similarly, a recent study reported that RIPK1/3 and MLKL were increased in CS-induced murine experimental COPD. pRIPK3 and pMLKL were also more increased in the lung tissues of patients with severe COPD than in those of nonsmokers or non-COPD smokers. Importantly, cellular and molecular airway inflammations were reduced in RIPK3^−/−^ and MLKL^−/−^ mice. MLKL^−/−^ mice also had a suppressed airway remodeling and emphysema, but no treatment inhibited the apoptosis (pan-caspase inhibitor qVD-OPh) (Lu et al., 2021). Another recent study also reported the same conclusion that CS-induced necroptosis contributes to the pathogenesis of COPD. However, Nec-1 treatment or RIPK1 silencing by siRNA did not protect against CS-induced emphysema and or suppress the lung inflammation in mice, while RIPK3 inhibitor GSK’872 had the protective effect (Chen et al., 2021). Moreover, airborne PM exposure can trigger necroptosis in human bronchial epithelial (HBE) cells or mouse airways, which is involved in the pathogenesis of PM-induced pulmonary inflammation and mucus hyperproduction reduced by Nec-1 and GSK’872 (Peixoto et al., 2017; Xu et al., 2018). Overall, RIPK3-MLKL-dependent necroptosis plays an important role in COPD pathogenesis.

Mitochondria-specific autophagy (mitophagy) plays an important physiological role in maintaining a healthy and functional mitochondrial network (Chen et al., 2008; Pehote and Vij, 2020; Vishnupriya et al., 2020). However, excessive autophagy activation can refer to the pathological role of alveolar epithelial cells and mitophagy is a possible pathogenic mediator of COPD (Mizumura et al., 2012). In addition, CS exposure has been found to cause mitophagy and mitochondrial dysfunction. Interestingly, an emerging hypothesis states that CS-induced mitophagy is involved in necroptosis in pulmonary epithelial cells and murine models (Mizumura et al., 2014). A study reported that the expression level of PTEN-induced kinase 1 (PINK1) and RIPK3 was increased in human epithelial cells with COPD. However, CS-induced cell death, mitochondrial dysfunction, and MLKL phosphorylation are blocked in PINK1-knockdown cells and mitophagy inhibitor Mdivi-1-treated Beas-2B cells (Mizumura et al., 2014). In human lung epithelial and endothelial cells, CS exposure triggers necroptosis, which requires the stabilization of PINK1 with mitophagy through a mechanism involving the excessive accumulation of palmitoyl (C16)-ceramide (Cer), which is an important mediator. High lignoceroyl (C24)-dihydroceramide levels may protect against CS-induced necroptosis. In conclusion, PINK1-regulated lethal mitophagy and mitophagy-mediated necroptosis both contribute to COPD, in which C16-Cer could be an upstream initiator (Mizumura et al., 2018). Further studies are needed to clarify the role and mechanism of CS-induced necroptosis in lung.

### Idiopathic Pulmonary Fibrosis

Particulate inhalation, genetic susceptibility, and CS play a role in IPF pathogenesis and progression. IPF is characterized by type 1/2 alveolar epithelial cell (AEC1/AEC2) injury and failure to repair, with the consequential activation of fibroblast/myofibroblasts that destroy normal alveolar architecture (Richeldi et al., 2017). Necroptosis is believed to be implicated in IPF development. In the lung tissue of patients with IPF, the level of RIPK3 expression is increased, indicating the involvement of necroptosis in IPF. RIPK3, HMGB1, and IL-1β levels have been reported to increase in bleomycin-induced IPF model; however, these levels were reduced in RIPK3-knockout mice and by Nec-1 that exhibits an inhibitory effect on inflammation and fibrosis (Lee et al., 2018). Mutation in SFTPA1 resulted in IPF in a consanguineous Japanese family, and SFTPA1 knock-in (Sftpa1-KI) mice spontaneously developed pulmonary fibrosis with increased necroptosis derived by c-Jun N-terminal kinase (JNK)-mediated upregulation of RIPK3 in AEC2s. JNK inhibition ameliorated pulmonary fibrosis in Sftpa1-KI mice, but it was blocked with RIPK3 overexpression (Takezaki et al., 2019).

Interestingly, IPF and COPD have a lot in common in terms of pathogenesis, including their relationship with smoking, lung aging biopathological processes (Duckworth et al., 2021; Schuliga et al., 2021) and necroptosis-involved pathological mechanisms. A study reported that CS aggravates bleomycin-induced pulmonary fibrosis via TGF-β1 signaling, but necroptosis was not mentioned (Zhou et al., 2019). It will be interesting to explore the function of necroptosis in CS-related pulmonary fibrosis and determine if CS-induced necroptosis leads to IPF or COPD.

### Pulmonary Arterial Hypertension

PAH is a progressive cardiopulmonary disease characterized by perivascular infiltration by inflammatory cells, adverse vascular remodeling, vascular fibrosis, and stiffening. Some preclinical studies have shown that inflammation plays a pathogenic role in PAH development and advanced vascular remodeling may be reversed by approaches addressing specific inflammatory and immune processes (Rabinovitch et al., 2014). Evidence showing that necroptosis is implicated in PAH is limited. HMGB1, one of the DAMPs, plays a crucial role in the development of PAH and manifestation of sex difference in PAH severity. Males are prone to show a more progressive and severe PAH development with a higher level of circulating HMGB1, which may mediate downstream signaling through TLR4 activation. Necroptosis and necrosis are the primary sources of circulating HMGB1 in male rats, whereas only the attenuation of necrosis prevents TLR4 activation and blunts the sex differences in PAH severity. Collectively, necroptosis and necrosis play a potential role in HMGB1 release, TLR4 activation, and sex difference manifestation in PAH severity (Zemskova et al., 2020). Similarly, in a rat model of monocrotaline-induced PAH, bioinformatics analysis revealed that RIPK3-mediated necroptosis is involved in the generation of DAMPs that are associated with the activation of TLR and NOD-like receptor pathways (Xiao et al., 2020).

### Lung Cancer

Apoptosis plays a well-recognized role in defenses against tumors, whereas the evasion of and resistance to apoptosis is often responsible for both tumorigenesis and chemotherapeutic drug resistance (Hanahan and Weinberg, 2011; Gong et al., 2019). The role of necroptosis in tumors is complicated, involving tumorigenesis and malignant progression, promoting tumor metastasis and drug resistance. Evidence shows that tumors induce endothelial cell necroptosis, leading to extravasation and metastasis via the amyloid precursor protein and its receptor, death receptor 6, which is expressed on endothelial cells as a primary mediator. These effects can be blocked with Nec-1 or RIPK3 deletion (Strilic et al., 2016). Similarly, TAK1 plays an inhibitory role in endothelial necroptosis and metastasis. TAK1 deficiency is more likely to cause RIPK3-dependent necroptosis of human/murine endothelial cells by upregulating RIPK3 expression and form metastases by the endothelium (Yang et al., 2019). However, necroptosis may trigger and amplify antitumor immunity in cancer therapy by eliciting strong adaptive immune responses to defend against tumor progression (Su et al., 2016; Gong et al., 2019). Several studies have reported that various cancer cells can undergo necroptosis due to necroptosis inducers and chemotherapeutic agents (Table 2). An interesting study has also identified that many cancer cells have intrinsic or acquired defects in the mechanism of necroptosis (Su et al., 2016). RIPK3, the key regulatory factor of necroptosis, shows the potential for predicting response after treatment. In one situation, a high level of RIPK3 was associated with improved local control and progression-free survival in patients with non-small cell lung cancer (NSCLC) after hypofractionated radiation therapy (Wang HH. et al., 2018). In another situation, low RIPK3 showed worse disease-free survival (DFS) after curative resection in patients with NSCLC (Park et al., 2020). Similarly, patients with NSCLC who have lower RIPK3 expression have worse chemotherapy responses (Wang Q. et al., 2020). Thus, RIPK3-mediated necroptosis pathway may be suppressed in lung cancer cells and lose its antitumor function. However, a study with 404 patients of NSCLC found that a higher RIPK3 expression is associated with shorter overall survival and a tendency of shorter DFS, which might be caused by the resistance to radiotherapy or excessive necroptosis-mediated damage (Kim et al., 2020). Nevertheless, necroptosis-based antitumor may be a promising therapeutic strategy worthy of further investigation. A better understanding of the role of necroptosis in lung cancer is expected to exploit necroptosis for lung cancer therapies. Certainly, concerns such as how to specifically induce the necroptosis of cancer cells, whether necroptosis-based antitumor has a deleterious role, and how to overcome necroptosis resistance in cancer cells still exist (Su et al., 2016; Gong et al., 2019).

**TABLE 2:** Substances that have an inhibitory effect on tumors by mediating necroptosis.

Classification	Name	Source	Mechanism
**Natural compounds or extracts**	2-methoxy-6-acetyl-7methyljuglone (MAM) (Sun et al., 2016; Sun et al., 2019b)	A naphthoquinone isolated from polygonum cuspidatum	Induce NO-dependent necroptosis or apoptosis mediated by H2O2-dependent JNK activation in cancer cells
	Tanshinol A (TSA) (Liu et al., 2020a)	A tanshinone that isolated from the roots of Danshen	Trigger MLKL-dependent necroptosis that independent RIPK1/RIPK3 and calcium. ROS might promote the upstream of MLKL.
	sea hare hydrolysates (SHH) (Nyiramana et al., 2020)	A hydrolysate from Sea Hare	SHH has effects against lung cancer by activating M1 (as anti-tumor effects), reducing M2, inhibiting growth and migration, and being cytotoxicity. Pyroptosis/necroptosis take part in SHH-induced anticancer effects under STAT3 inhibition
	Shikonin (Kim et al., 2017)	Purified from lithospermum erythrorhizon	Induce necroptosis and autophagy in NSCLC cells. Necroptosis is enhanced by inhibition of shikonin-induced autophagy
	Citronellol (Yu et al., 2019)	A monoterpene having the molecular formula of C10H20O	Induce necroptosis of NCI-H1299 cells by TNF- α pathway and ROS accumulation
**Substances in tumor cells**	Betanodavirus B2 protein (Chiu et al., 2017)	Encoded by a sub-genomic RNA3 in betanodaviruses replication	Trigger apoptosis required P53 activation and RIPK3-dependent necroptosis by ROS
	Sirtuin (SIRT3) (Tang et al., 2020)	A member of the Sirtuin family of nicotinamide adenine dinucleotide (NAD+)-dependent deacetylases	Keep mutant p53 stable by controlling proteasomal degradation triggered by ubiquitylation and has anti-tumor effects by inducing apoptosis and necroptosis
	Kras-derived exosomes (Petanidis et al., 2020)	A exosomes	Treatment with carboplatin and inhibition of Kras secreted exosomes induce TNFα-mediated RIPK3-dependent necroptosis and reduce miR-146/miR-210 levels that have the effects of immunosuppressive. Reduction in miR-146/miR-210 levels accompanied by a reduction in immunosuppressive
	PITPα(Jing et al., 2018)	A family member of PITPs	Promote cisplatin-induced MLKL-dependent necroptosis by increasing oligomerization and plasma membrane translocation
**Synthetic compound**	3-bromomethylbenzofuran-2-carbox-ylic acid ethyl ester (MCC1019) (Abdelfatah et al., 2019)	Drug-like compounds	Suppress AKT signaling pathway activation. prolong mitotic arrest and induced apoptosis and necroptosis
	2-amino-2-[2-(4-octylphenyl)ethyl] propane-1,3-diol; Fingolimod, Novartis ( FTY720) (Saddoughi et al., 2013)	A synthetic sphingosine analogue of myriocin	Binds I2PP2A/SET then activate PP2A tumor suppressor signaling, and induce RIPK1-mediated necroptosis
	LGH00168 (Ma et al., 2016)	Drug-like compounds, C/EBP homologous protein (CHOP) activator	A CHOP activator that induces necroptosis by ROS-mediated ER stress, CHOP activation, and NF-kB inhibition
	ethyl 6-(5-(phenylsulfonamido)pyridin-3y-l)imidazo [1,2a]pyridine-3-carboxylate (HS-173) (Park et al., 2019)	The imidazopyridine derivative	Induce necroptosis by enhancing RIPK3 expression and activating the RIPK3/MLKL signaling pathway in lung cancer cells
	PK68(Hou et al., 2019)	A potent and selective type II RIPK1 inhibitor	Prophylactic use of PK68 has an inhibitory effect on tumor metastasis

## Opportunities for Therapy

Collectively, necroptosis is closely related to multiple human pathologies and inappropriate necroptosis causes excessive immune or inflammatory responses and tissue damage, leading to condition deterioration. Clearly, interrupting the necroptosis pathway by inhibitors will exert strong beneficial effects. Few trials show that inhibitors of necroptosis-related molecules were being developed for the treatment of psoriasis, rheumatoid arthritis, ulcerative colitis, and other inflammatory diseases or cancer (Fang et al., 2021).

GSK2982772 is a RIPK1 inhibitor. The first-in-human study showed that single and repeat doses of GSK2982772 were generally safe and well-tolerated in healthy adult volunteers, which support progression into Phase II clinical trials (Weisel et al., 2017; Tompson et al., 2021). The phase 2a clinical trials showed a certain therapeutic effect on active plaque psoriasis, and other phase 2a clinical trials in moderate to severe psoriasis are currently underway (Weisel et al., 2020). However, the phase 2a clinical trials showed GSK2982772 have no effects in severe rheumatoid arthritis and active ulcerative colitis (Weisel et al., 2021a; Weisel et al., 2021b). Different effects may be related to the type of disease or due to the small number of patients included in the trials.

DNL104, a selective centrally penetrant RIPK1 inhibitor, is generally safe and well-tolerated in the clinical development for Alzheimer’s disease and amyotrophic lateral sclerosis in a randomized phase I ascending dose study in healthy volunteers (Grievink et al., 2020). Interestingly, some US Food and Drug Administration-approved anticancer drugs, including ponatinib and pazopanib, can inhibit RIPK1; these drugs would be better prospects in further clinical studies (Fauster et al., 2015). However, there is only limited evidence regarding the inhibitors of necroptosis-related molecules for the treatment of pulmonary diseases. More inhibitors that are suitable for advancement into the clinic have yet to be described.

## Conclusion

Necroptosis, as a major pathway of RCD, is important in the embryonic and postnatal development and causes an innate immune response to protect the body, especially in cases of viral infections and tumors. Increasing evidences shows that necroptosis is a possible target for the treatment of pulmonary diseases in the future.

However, many questions are yet to be addressed. The establishment of specific, sensitive, and reliable molecular markers of necroptosis is important because necroptosis-relevant proteins have a variety of functions, including RCD-unrelated functions. The correlation among various RCDs, extent to which necroptosis and apoptosis pathways are different, and connection mechanism between two cell deaths remain unclear. Moreover, studying how necroptosis connects to other biological processes is conducive to the development of targeted drugs for necroptosis without overreaction or sequela. Most importantly, more clinical trials are needed to confirm the roles of necroptosis inhibitors or other necroptosis-targeting drugs. In addition, targeting immune enhancement and tumor suppression may be the future direction of treatment. The effects and side effects of necroptosis-targeting drugs should be studied further in the future.

## References

[B1] Abdelfatah S. Berg A. Huang Q. Yang L. J. Hamdoun S. Klinger A. (2019). MCC1019, a Selective Inhibitor of the Polo-box Domain of Polo-like Kinase 1 as Novel, Potent Anticancer Candidate. Acta Pharm. Sin B 9 (5), 1021–1034. 10.1016/j.apsb.2019.02.001 31649851 PMC6804483

[B2] Anderton H. Bandala-Sanchez E. Simpson D. S. Rickard J. A. Ng A. P. Di Rago L. (2019). RIPK1 Prevents TRADD-Driven, but TNFR1 Independent, Apoptosis during Development. Cell Death Differ 26 (5), 877–889. 10.1038/s41418-018-0166-8 30185824 PMC6461919

[B3] Bedient L. Pokharel S. M. Chiok K. R. Mohanty I. Beach S. S. Miura T. A. (2020). Lytic Cell Death Mechanisms in Human Respiratory Syncytial Virus-Infected Macrophages: Roles of Pyroptosis and Necroptosis. Viruses 12 (9), 932. 10.3390/v12090932 PMC7552060 32854254

[B4] Bolognese A. C. Yang W.-L. Hansen L. W. Denning N.-L. Nicastro J. M. Coppa G. F. (2018). Inhibition of Necroptosis Attenuates Lung Injury and Improves Survival in Neonatal Sepsis. Surgery 164, 110–116. 10.1016/j.surg.2018.02.017 PMC6204110 29709367

[B5] Brightling C. Berry M. Amrani Y. (2008). Targeting TNF-Alpha: a Novel Therapeutic Approach for Asthma. J. Allergy Clin. Immunol. 121 (1), 5–2. 10.1016/j.jaci.2007.10.028 18036647 PMC3992375

[B6] Butler R. E. Krishnan N. Garcia-Jimenez W. Francis R. Martyn A. Mendum T. (2017). Susceptibility of Mycobacterium Tuberculosis-Infected Host Cells to Phospho-MLKL Driven Necroptosis Is Dependent on Cell Type and Presence of TNFα. Virulence 8 (8), 1820–1832. 10.1080/21505594.2017.1377881 28892415 PMC5750806

[B7] Carnino J. M. Lee H. He X. Groot M. Jin Y. (2020). Extracellular Vesicle-Cargo miR-185-5p Reflects Type II Alveolar Cell Death after Oxidative Stress. Cell Death Discov 6, 82. 10.1038/s41420-020-00317-8 PMC7484781 32963810

[B8] Cerps S. C. Menzel M. Mahmutovic Persson I. Bjermer L. Akbarshahi H. Uller L. (2018). Interferon-β Deficiency at Asthma Exacerbation Promotes MLKL Mediated Necroptosis. Sci. Rep. 8 (1), 4248. 10.1038/s41598-018-22557-6 29523863 PMC5844912

[B9] Chen D. Gregory A. D. Li X. Wei J. Burton C. L. Gibson G. (2021). RIP3-dependent Necroptosis Contributes to the Pathogenesis of Chronic Obstructive Pulmonary Disease. JCI Insight 6 (12), e144689. 10.1172/jci.insight.144689 PMC8262480 34156033

[B10] Chen J. Wang S. Fu R. Zhou M. Zhang T. Pan W. (2018). RIP3 Dependent NLRP3 Inflammasome Activation Is Implicated in Acute Lung Injury in Mice. J. Transl Med. 16 (1), 233. 10.1186/s12967-018-1606-4 30126430 PMC6102827

[B11] Chen Z. H. Kim H. P. Sciurba F. C. Lee S. J. Feghali-Bostwick C. Stolz D. B. (2008). Egr-1 Regulates Autophagy in Cigarette Smoke-Induced Chronic Obstructive Pulmonary Disease. PLoS One 3 (10), e3316. 10.1371/journal.pone.0003316 18830406 PMC2552992

[B12] Chiu H. W. Su Y. C. Hong J. R. (2017). Betanodavirus B2 Protein Triggers Apoptosis and Necroptosis in Lung Cancer Cells that Suppresses Autophagy. Oncotarget 8 (55), 94129–94141. 10.18632/oncotarget.21588 29212215 PMC5706861

[B13] Cho Y. S. Challa S. Moquin D. Genga R. Ray T. D. Guildford M. (2009). Phosphorylation-driven Assembly of the RIP1-RIP3 Complex Regulates Programmed Necrosis and Virus-Induced Inflammation. Cell 137 (6), 1112–1123. 10.1016/j.cell.2009.05.037 19524513 PMC2727676

[B14] Choi M. E. Price D. R. Ryter S. W. Choi A. M. K. (2019). Necroptosis: a Crucial Pathogenic Mediator of Human Disease. JCI Insight 4 (15), e128834. 10.1172/jci.insight.128834 PMC6693822 31391333

[B15] Conos S. A. Chen K. W. De Nardo D. Hara H. Whitehead L. Núñez G. (2017). Active MLKL Triggers the NLRP3 Inflammasome in a Cell-Intrinsic Manner. Proc. Natl. Acad. Sci. U S A. 114 (6), E961–E969. 10.1073/pnas.1613305114 28096356 PMC5307433

[B16] Cook W. D. Moujalled D. M. Ralph T. J. Lock P. Young S. N. Murphy J. M. (2014). RIPK1- and RIPK3-Induced Cell Death Mode Is Determined by Target Availability. Cell Death Differ 21 (10), 1600–1612. 10.1038/cdd.2014.70 24902899 PMC4158685

[B17] Daley-Bauer L. P. Roback L. Crosby L. N. McCormick A. L. Feng Y. Kaiser W. J. (2017). Mouse Cytomegalovirus M36 and M45 Death Suppressors Cooperate to Prevent Inflammation Resulting from Antiviral Programmed Cell Death Pathways. Proc. Natl. Acad. Sci. U S A. 114 (13), E2786–E2795. 10.1073/pnas.1616829114 28292903 PMC5380087

[B18] Daniels B. P. Snyder A. G. Olsen T. M. Orozco S. Oguin T. H. 3rd Tait S. W. G. (2017). RIPK3 Restricts Viral Pathogenesis via Cell Death-independent Neuroinflammation. Cell 169 (2), 301. 10.1016/j.cell.2017.03.011 28366204 PMC5405738

[B19] Degterev A. Hitomi J. Germscheid M. Ch'en I. L. Korkina O. Teng X. (2008). Identification of RIP1 Kinase as a Specific Cellular Target of Necrostatins. Nat. Chem. Biol. 4 (5), 313–321. 10.1038/nchembio.83 18408713 PMC5434866

[B20] Degterev A. Huang Z. Boyce M. Li Y. Jagtap P. Mizushima N. (2005). Chemical Inhibitor of Nonapoptotic Cell Death with Therapeutic Potential for Ischemic Brain Injury. Nat. Chem. Biol. 1 (2), 112–119. 10.1038/nchembio711 16408008

[B21] Dillon C. P. Weinlich R. Rodriguez D. A. Cripps J. G. Quarato G. Gurung P. (2014). RIPK1 Blocks Early Postnatal Lethality Mediated by Caspase-8 and RIPK3. Cell 157 (5), 1189–1202. 10.1016/j.cell.2014.04.018 24813850 PMC4068710

[B22] Dondelinger Y. Jouan-Lanhouet S. Divert T. Theatre E. Bertin J. Gough P. J. (2015). NF-κB-Independent Role of IKKα/IKKβ in Preventing RIPK1 Kinase-dependent Apoptotic and Necroptotic Cell Death during TNF Signaling. Mol. Cell 60 (1), 63–76. 10.1016/j.molcel.2015.07.032 26344099

[B23] Downey J. Pernet E. Coulombe F. Allard B. Meunier I. Jaworska J. (2017). RIPK3 Interacts with MAVS to Regulate Type I IFN-Mediated Immunity to Influenza A Virus Infection. Plos Pathog. 13 (4), e1006326. 10.1371/journal.ppat.1006326 28410401 PMC5406035

[B24] Du X. K. Ge W. Y. Jing R. Pan L. H. (2019). Necroptosis in Pulmonary Macrophages Mediates Lipopolysaccharide-Induced Lung Inflammatory Injury by Activating ZBP-1. Int. Immunopharmacol 77, 105944. 10.1016/j.intimp.2019.105944 31655343

[B25] Duckworth A. Gibbons M. A. Allen R. J. Almond H. Beaumont R. N. Wood A. R. (2021). Telomere Length and Risk of Idiopathic Pulmonary Fibrosis and Chronic Obstructive Pulmonary Disease: a Mendelian Randomisation Study. Lancet Respir. Med. 9 (3), 285–294. 10.1016/S2213-2600(20)30364-7 33197388

[B26] Duprez L. Takahashi N. Van Hauwermeiren F. Vandendriessche B. Goossens V. Vanden Berghe T. (2011). RIP Kinase-dependent Necrosis Drives Lethal Systemic Inflammatory Response Syndrome. Immunity 35 (6), 908–918. 10.1016/j.immuni.2011.09.020 22195746

[B27] Eng S. S. DeFelice M. L. (2016). The Role and Immunobiology of Eosinophils in the Respiratory System: a Comprehensive Review. Clin. Rev. Allergy Immunol. 50 (2), 140–158. 10.1007/s12016-015-8526-3 26797962

[B28] Fan E. K. Y. Fan J. (2018). Regulation of Alveolar Macrophage Death in Acute Lung Inflammation. Respir. Res. 19 (1), 50. 10.1186/s12931-018-0756-5 29587748 PMC5872399

[B29] Fang Z. Wei H. Gou W. Chen L. Bi C. Hou W. (2021). Recent Progress in Small-Molecule Inhibitors for Critical Therapeutic Targets of Necroptosis. Future Med. Chem. 13 (9), 817–837. 10.4155/fmc-2020-0386 33845591

[B30] Faust H. Lam L. M. Hotz M. J. Qing D. Mangalmurti N. S. (2020). RAGE Interacts with the Necroptotic Protein RIPK3 and Mediates Transfusion-Induced Danger Signal Release. Vox Sang 115 (8), 729–734. 10.1111/vox.12946 32633835 PMC8215843

[B31] Fauster A. Rebsamen M. Huber K. V. Bigenzahn J. W. Stukalov A. Lardeau C. H. (2015). A Cellular Screen Identifies Ponatinib and Pazopanib as Inhibitors of Necroptosis. Cell Death Dis 6 (5), e1767. 10.1038/cddis.2015.130 25996294 PMC4669708

[B32] Feng L. Yin Y. Y. Liu C. H. Xu K. R. Li Q. R. Wu J. R. (2020). Proteome-wide Data Analysis Reveals Tissue-specific Network Associated with SARS-CoV-2 Infection. J. Mol. Cell Biol 12 (12), 946–957. 10.1093/jmcb/mjaa033 32642770 PMC7454804

[B33] Frank D. Vince J. E. (2019). Pyroptosis versus Necroptosis: Similarities, Differences, and Crosstalk. Cell Death Differ 26 (1), 99–114. 10.1038/s41418-018-0212-6 30341423 PMC6294779

[B34] Fritsch M. Günther S. D. Schwarzer R. Albert M. C. Schorn F. Werthenbach J. P. (2019). Caspase-8 Is the Molecular Switch for Apoptosis, Necroptosis and Pyroptosis. Nature 575 (7784), 683–687. 10.1038/s41586-019-1770-6 31748744

[B35] Fuchs Y. Steller H. (2015). Live to die another way: modes of programmed cell death and the signals emanating from dying cells. Nat. Rev. Mol. Cell Biol 16 (6), 329–344. 10.1038/nrm3999 25991373 PMC4511109

[B36] Galluzzi L. Vitale I. Aaronson S. A. Abrams J. M. Adam D. Agostinis P. (2018). Molecular Mechanisms of Cell Death: Recommendations of the Nomenclature Committee on Cell Death 2018. Cell Death Differ 25 (3), 486–541. 10.1038/s41418-017-0012-4 29362479 PMC5864239

[B37] Garcia L. R. Tenev T. Newman R. Haich R. O. Liccardi G. John S. W. (2021). Ubiquitylation of MLKL at Lysine 219 Positively Regulates Necroptosis-Induced Tissue Injury and Pathogen Clearance. Nat. Commun. 12 (1), 3364. 10.1038/s41467-021-23474-5 34099649 PMC8184782

[B38] Garnish S. E. Meng Y. Koide A. Sandow J. J. Denbaum E. Jacobsen A. V. (2021). Conformational Interconversion of MLKL and Disengagement from RIPK3 Precede Cell Death by Necroptosis. Nat. Commun. 12 (1), 2211. 10.1038/s41467-021-22400-z 33850121 PMC8044208

[B39] Geng J. Ito Y. Shi L. Amin P. Chu J. Ouchida A. T. (2017). Regulation of RIPK1 Activation by TAK1-Mediated Phosphorylation Dictates Apoptosis and Necroptosis. Nat. Commun. 8 (1), 359. 10.1038/s41467-017-00406-w 28842570 PMC5572456

[B40] Ghimire L. Paudel S. Jin L. Baral P. Cai S. Jeyaseelan S. (2018). NLRP6 Negatively Regulates Pulmonary Host Defense in Gram-Positive Bacterial Infection through Modulating Neutrophil Recruitment and Function. Plos Pathog. 14 (9), e1007308. 10.1371/journal.ppat.1007308 30248149 PMC6171945

[B41] Gong T. Liu L. Jiang W. Zhou R. (2020). DAMP-sensing Receptors in Sterile Inflammation and Inflammatory Diseases. Nat. Rev. Immunol. 20 (2), 95–112. 10.1038/s41577-019-0215-7 31558839

[B42] Gong Y. Fan Z. Luo G. Yang C. Huang Q. Fan K. (2019). The Role of Necroptosis in Cancer Biology and Therapy. Mol. Cancer 18 (1), 100. 10.1186/s12943-019-1029-8 31122251 PMC6532150

[B43] Gong Y. N. Guy C. Olauson H. Becker J. U. Yang M. Fitzgerald P. (2017). ESCRT-III Acts Downstream of MLKL to Regulate Necroptotic Cell Death and its Consequences. Cell 169 (2), 286. 10.1016/j.cell.2017.03.020 28388412 PMC5443414

[B44] Gonzalez-Juarbe N. Bradley K. M. Riegler A. N. Reyes L. F. Brissac T. Park S. S. (2018). Bacterial Pore-Forming Toxins Promote the Activation of Caspases in Parallel to Necroptosis to Enhance Alarmin Release and Inflammation during Pneumonia. Sci. Rep. 8 (1), 5846. 10.1038/s41598-018-24210-8 29643440 PMC5895757

[B45] González-Juarbe N. Bradley K. M. Shenoy A. T. Gilley R. P. Reyes L. F. Hinojosa C. A. (2017). Pore-forming Toxin-Mediated Ion Dysregulation Leads to Death Receptor-independent Necroptosis of Lung Epithelial Cells during Bacterial Pneumonia. Cell Death Differ 24 (5), 917–928. 10.1038/cdd.2017.49 28387756 PMC5423117

[B46] González-Juarbe N. Gilley R. P. Hinojosa C. A. Bradley K. M. Kamei A. Gao G. (2015). Pore-Forming Toxins Induce Macrophage Necroptosis during Acute Bacterial Pneumonia. Plos Pathog. 11 (12), e1005337. 10.1371/journal.ppat.1005337 26659062 PMC4676650

[B47] Gonzalez-Juarbe N. Riegler A. N. Jureka A. S. Gilley R. P. Brand J. D. Trombley J. E. (2020). Influenza-Induced Oxidative Stress Sensitizes Lung Cells to Bacterial-Toxin-Mediated Necroptosis. Cell Rep 32 (8), 108062. 10.1016/j.celrep.2020.108062 32846120 PMC7570217

[B48] Grievink H. W. Heuberger J. A. A. C. Huang F. Chaudhary R. Birkhoff W. A. J. Tonn G. R. (2020). DNL104, a Centrally Penetrant RIPK1 Inhibitor, Inhibits RIP1 Kinase Phosphorylation in a Randomized Phase I Ascending Dose Study in Healthy Volunteers. Clin. Pharmacol. Ther. 107 (2), 406–414. 10.1002/cpt.1615 31437302

[B49] Hakansson A. P. Bergenfelz C. (2017). Low NF-Κb Activation and Necroptosis in Alveolar Macrophages: A New Virulence Property of Streptococcus Pneumoniae. J. Infect Dis. 216 (4), 402–404. 10.1093/infdis/jix161 28368457

[B50] Han C. H. Guan Z. B. Zhang P. X. Fang H. L. Li L. Zhang H. M. (2018). Oxidative Stress Induced Necroptosis Activation Is Involved in the Pathogenesis of Hyperoxic Acute Lung Injury. Biochem. Biophys. Res. Commun. 495 (3), 2178–2183. 10.1016/j.bbrc.2017.12.100 29269294

[B51] Hanahan D. Weinberg R. A. (2011). Hallmarks of Cancer: the Next Generation. Cell 144 (5), 646–674. 10.1016/j.cell.2011.02.013 21376230

[B52] He S. Liang Y. Shao F. Wang X. (2011). Toll-like Receptors Activate Programmed Necrosis in Macrophages through a Receptor-Interacting Kinase-3-Mediated Pathway. Proc. Natl. Acad. Sci. U S A. 108 (50), 20054–20059. 10.1073/pnas.1116302108 22123964 PMC3250173

[B53] He S. Wang L. Miao L. Wang T. Du F. Zhao L. (2009). Receptor Interacting Protein Kinase-3 Determines Cellular Necrotic Response to TNF-Alpha. Cell 137 (6), 1100–1111. 10.1016/j.cell.2009.05.021 19524512

[B54] He S. Wang X. (2018). RIP Kinases as Modulators of Inflammation and Immunity. Nat. Immunol. 19 (9), 912–922. 10.1038/s41590-018-0188-x 30131615

[B55] Hou J. Ju J. Zhang Z. Zhao C. Li Z. Zheng J. (2019). Discovery of Potent Necroptosis Inhibitors Targeting RIPK1 Kinase Activity for the Treatment of Inflammatory Disorder and Cancer Metastasis. Cell Death Dis 10 (7), 493. 10.1038/s41419-019-1735-6 31235688 PMC6591251

[B56] Huang X. Tan S. Li Y. Cao S. Li X. Pan H. (2021). Caspase Inhibition Prolongs Inflammation by Promoting a Signaling Complex with Activated RIPK1. J. Cell Biol 220 (6), e202007127. 10.1083/jcb.202007127 33914027 PMC8091129

[B57] Iyer S. S. Pulskens W. P. Sadler J. J. Butter L. M. Teske G. J. Ulland T. K. (2009). Necrotic Cells Trigger a Sterile Inflammatory Response through the Nlrp3 Inflammasome. Proc. Natl. Acad. Sci. U S A. 106 (48), 20388–20393. 10.1073/pnas.0908698106 19918053 PMC2787135

[B58] Jaco I. Annibaldi A. Lalaoui N. Wilson R. Tenev T. Laurien L. (2017). MK2 Phosphorylates RIPK1 to Prevent TNF-Induced Cell Death. Mol. Cell 66 (5), 698. 10.1016/j.molcel.2017.05.003 28506461 PMC5459754

[B59] Jiao H. Wachsmuth L. Kumari S. Schwarzer R. Lin J. Eren R. O. (2020). Z-nucleic-acid Sensing Triggers ZBP1-dependent Necroptosis and Inflammation. Nature 580 (7803), 391–395. 10.1038/s41586-020-2129-8 32296175 PMC7279955

[B60] Jing L. Song F. Liu Z. Li J. Wu B. Fu Z. (2018). MLKL-PITPα Signaling-Mediated Necroptosis Contributes to Cisplatin-Triggered Cell Death in Lung Cancer A549 Cells. Cancer Lett. 414, 136–146. 10.1016/j.canlet.2017.10.047 29104146

[B61] Jondle C. N. Gupta K. Mishra B. B. Sharma J. (2018). Klebsiella pneumoniae Infection of Murine Neutrophils Impairs Their Efferocytic Clearance by Modulating Cell Death Machinery. Plos Pathog. 14 (10), e1007338. 10.1371/journal.ppat.1007338 30273394 PMC6181436

[B62] Jose R. J. Manuel A. (2020). COVID-19 Cytokine Storm: the Interplay between Inflammation and Coagulation. Lancet Respir. Med. 8 (6), e46–e47. 10.1016/s2213-2600(20)30216-2 32353251 PMC7185942

[B63] Kaiser W. J. Sridharan H. Huang C. Mandal P. Upton J. W. Gough P. J. (2013). Toll-like Receptor 3-mediated Necrosis via TRIF, RIP3, and MLKL. J. Biol. Chem. 288 (43), 31268–31279. 10.1074/jbc.M113.462341 24019532 PMC3829437

[B64] Kanou T. Ohsumi A. Kim H. Chen M. Bai X. Guan Z. (2018). Inhibition of Regulated Necrosis Attenuates Receptor-Interacting Protein Kinase 1-mediated Ischemia-Reperfusion Injury after Lung Transplantation. J. Heart Lung Transpl. 37 (10), 1261–1270. 10.1016/j.healun.2018.04.005 29907500

[B65] Karki R. Sharma B. R. Tuladhar S. Williams E. P. Zalduondo L. Samir P. (2021). Synergism of TNF-α and IFN-γ Triggers Inflammatory Cell Death, Tissue Damage, and Mortality in SARS-CoV-2 Infection and Cytokine Shock Syndromes. Cell 184 (1), 149–168. 10.1016/j.cell.2020.11.025 33278357 PMC7674074

[B66] Kaup F. J. Drommer W. Damsch S. Deegen E. (1990). Ultrastructural Findings in Horses with Chronic Obstructive Pulmonary Disease (COPD). II: Pathomorphological Changes of the Terminal Airways and the Alveolar Region. Equine Vet. J. 22 (5), 349–355. 10.1111/j.2042-3306.1990.tb04288.x 2226400

[B67] Kim H. Zamel R. Bai X. H. Lu C. Keshavjee S. Keshavjee S. (2018). Ischemia-reperfusion Induces Death Receptor-independent Necroptosis via Calpain-STAT3 Activation in a Lung Transplant Setting. Am. J. Physiol. Lung Cell Mol PhysiolLung Cell. Mol. Physiol. 315 (4), L595–L608. 10.1152/ajplung.00069.2018 30024306

[B68] Kim H. J. Hwang K. E. Park D. S. Oh S. H. Jun H. Y. Yoon K. H. (2017). Shikonin-induced Necroptosis Is Enhanced by the Inhibition of Autophagy in Non-small Cell Lung Cancer Cells. J. Transl Med. 15 (1), 123. 10.1186/s12967-017-1223-7 28569199 PMC5452303

[B69] Kim J. Chung J. Y. Park Y. S. Jang S. J. Kim H. R. Choi C. M. (2020). Prognostic Significance of CHIP and RIPK3 in Non-small Cell Lung Cancer. Cancers (Basel) 12 (6), 1496. 10.3390/cancers12061496 PMC7352347 32521727

[B70] Kishino A. Hayashi K. Maeda M. Jike T. Hidai C. Nomura Y. (2019). Caspase-8 Regulates Endoplasmic Reticulum Stress-Induced Necroptosis Independent of the Apoptosis Pathway in Auditory Cells. Int. J. Mol. Sci. 20 (23), 5896. 10.3390/ijms20235896 PMC6928907 31771290

[B71] Kitur K. Parker D. Nieto P. Ahn D. S. Cohen T. S. Chung S. (2015). Toxin-induced Necroptosis Is a Major Mechanism of Staphylococcus aureus Lung Damage. Plos Pathog. 11 (4), e1004820. 10.1371/journal.ppat.1004820 25880560 PMC4399879

[B72] Koppe C. Verheugd P. Gautheron J. Reisinger F. Kreggenwinkel K. Roderburg C. (2016). IκB Kinaseα/β Control Biliary Homeostasis and Hepatocarcinogenesis in Mice by Phosphorylating the Cell-Death Mediator Receptor-Interacting Protein Kinase 1. Hepatology 64 (4), 1217–1231. 10.1002/hep.28723 27396433

[B73] Koupenova M. Corkrey H. A. Vitseva O. Tanriverdi K. Somasundaran M. Liu P. (2021). SARS-CoV-2 Initiates Programmed Cell Death in Platelets. Circ. Res. 129, 631–646. 10.1161/CIRCRESAHA.121.319117 34293929 PMC8409903

[B74] Lafont E. Draber P. Rieser E. Reichert M. Kupka S. de Miguel D. (2018). TBK1 and IKKε Prevent TNF-Induced Cell Death by RIPK1 Phosphorylation. Nat. Cell Biol 20 (12), 1389–1399. 10.1038/s41556-018-0229-6 30420664 PMC6268100

[B75] Lalaoui N. Boyden S. E. Oda H. Wood G. M. Stone D. L. Chau D. (2020). Mutations that Prevent Caspase Cleavage of RIPK1 Cause Autoinflammatory Disease. Nature 577 (7788), 103–108. 10.1038/s41586-019-1828-5 31827281 PMC6930849

[B76] Laurien L. Nagata M. Schünke H. Delanghe T. Wiederstein J. L. Kumari S. (2020). Autophosphorylation at Serine 166 Regulates RIP Kinase 1-mediated Cell Death and Inflammation. Nat. Commun. 11 (1), 1747. 10.1038/s41467-020-15466-8 32269263 PMC7142081

[B77] Lawlor K. E. Khan N. Mildenhall A. Gerlic M. Croker B. A. D'Cruz A. A. (2015). RIPK3 Promotes Cell Death and NLRP3 Inflammasome Activation in the Absence of MLKL. Nat. Commun. 6, 6282. 10.1038/ncomms7282 25693118 PMC4346630

[B78] Lee J. M. Yoshida M. Kim M. S. Lee J. H. Baek A. R. Jang A. S. (2018). Involvement of Alveolar Epithelial Cell Necroptosis in Idiopathic Pulmonary Fibrosis Pathogenesis. Am. J. Respir. Cell Mol Biol 59 (2), 215–224. 10.1165/rcmb.2017-0034OC 29444413

[B79] Li J. McQuade T. Siemer A. B. Napetschnig J. Moriwaki K. Hsiao Y. S. (2012). The RIP1/RIP3 Necrosome Forms a Functional Amyloid Signaling Complex Required for Programmed Necrosis. Cell 150 (2), 339–350. 10.1016/j.cell.2012.06.019 22817896 PMC3664196

[B80] Li S. Zhang Y. Guan Z. Li H. Ye M. Chen X. (2020a). SARS-CoV-2 Triggers Inflammatory Responses and Cell Death through Caspase-8 Activation. Signal Transduct Target Ther. 5 (1), 235. 10.1038/s41392-020-00334-0 33037188 PMC7545816

[B81] Li X. Chen M. Shi Q. Zhang H. Xu S. (2020b). Hydrogen Sulfide Exposure Induces Apoptosis and Necroptosis through lncRNA3037/miR-15a/BCL2-A20 Signaling in Broiler Trachea. Sci. Total Environ. 699, 134296. 10.1016/j.scitotenv.2019.134296 31683218

[B82] Li X. Gong W. Wang H. Li T. Attri K. S. Lewis R. E. (2019a). O-GlcNAc Transferase Suppresses Inflammation and Necroptosis by Targeting Receptor-Interacting Serine/Threonine-Protein Kinase 3. Immunity 50 (3), 1115. 10.1016/j.immuni.2019.03.008 30995496 PMC6508067

[B83] Li X. Yao X. Zhu Y. Zhang H. Wang H. Ma Q. (2019b). The Caspase Inhibitor Z-VAD-FMK Alleviates Endotoxic Shock via Inducing Macrophages Necroptosis and Promoting MDSCs-Mediated Inhibition of Macrophages Activation. Front Immunol. 10, 1824. 10.3389/fimmu.2019.01824 31428103 PMC6687755

[B84] Lin B. Jin Z. Chen X. Zhao L. Weng C. Chen B. (2020). Necrostatin-1 P-rotects M-ice from A-cute L-ung I-njury by S-uppressing N-ecroptosis and R-eactive O-xygen S-pecies. Mol. Med. Rep. 21 (5), 2171–2181. 10.3892/mmr.2020.11010 32323764 PMC7115190

[B85] Lin J. Kumari S. Kim C. Van T. M. Wachsmuth L. Polykratis A. (2016). RIPK1 Counteracts ZBP1-Mediated Necroptosis to Inhibit Inflammation. Nature 540 (7631), 124–128. 10.1038/nature20558 27819681 PMC5755685

[B86] Linkermann A. Green D. R. (2014). Necroptosis. N. Engl. J. Med. 370 (5), 455–465. 10.1056/NEJMra1310050 24476434 PMC4035222

[B87] Liu X. Zhang Y. Gao H. Hou Y. Lu J. J. Feng Y. (2020a). Induction of an MLKL Mediated Non-canonical Necroptosis through Reactive Oxygen Species by Tanshinol A in Lung Cancer Cells. Biochem. Pharmacol. 171, 113684. 10.1016/j.bcp.2019.113684 31678492

[B88] Liu Z. Fu Q. Tang S. Xie Y. Meng Q. Tang X. (2020b). Proteomics Analysis of Lung Reveals Inflammation and Cell Death Induced by Atmospheric H2S Exposure in Pig. Environ. Res. 191, 110204. 10.1016/j.envres.2020.110204 32937176

[B89] Lu Z. Van Eeckhoutte H. P. Liu G. Nair P. M. Jones B. Gillis C. M. (2021). Necroptosis Signalling Promotes Inflammation, Airway Remodelling and Emphysema in COPD. Am. J. Respir. Crit. Care Med. 10.1164/rccm.202009-3442OC 34133911

[B90] Ma Y. M. Peng Y. M. Zhu Q. H. Gao A. H. Chao B. He Q. J. (2016). Novel CHOP Activator LGH00168 Induces Necroptosis in A549 Human Lung Cancer Cells via ROS-Mediated ER Stress and NF-Κb Inhibition. Acta Pharmacol. Sin 37 (10), 1381–1390. 10.1038/aps.2016.61 27264312 PMC5057234

[B91] Mandal P. Berger S. B. Pillay S. Moriwaki K. Huang C. Guo H. (2014). RIP3 Induces Apoptosis Independent of Pronecrotic Kinase Activity. Mol. Cell 56 (4), 481–495. 10.1016/j.molcel.2014.10.021 25459880 PMC4512186

[B92] Martens S. Bridelance J. Roelandt R. Vandenabeele P. Takahashi N. (2021). MLKL in Cancer: More Than a Necroptosis Regulator. Cell Death Differ 28 (6), 1757–1772. 10.1038/s41418-021-00785-0 33953348 PMC8184805

[B93] Menon M. B. Gropengiesser J. Fischer J. Novikova L. Deuretzbacher A. Lafera J. (2017). p38MAPK/MK2-dependent Phosphorylation Controls Cytotoxic RIPK1 Signalling in Inflammation And infection. Nat. Cell Biol 19 (10), 1248–1259. 10.1038/ncb3614 28920954

[B94] Mizumura K. Cloonan S. M. Haspel J. A. Choi A. M. K. (2012). The Emerging Importance of Autophagy in Pulmonary Diseases. Chest 142 (5), 1289–1299. 10.1378/chest.12-0809 23131937 PMC3494477

[B95] Mizumura K. Cloonan S. M. Nakahira K. Bhashyam A. R. Cervo M. Kitada T. (2014). Mitophagy-dependent Necroptosis Contributes to the Pathogenesis of COPD. J. Clin. Invest 124 (9), 3987–4003. 10.1172/JCI74985 25083992 PMC4151233

[B96] Mizumura K. Justice M. J. Schweitzer K. S. Krishnan S. Bronova I. Berdyshev E. V. (2018). Sphingolipid Regulation of Lung Epithelial Cell Mitophagy and Necroptosis during Cigarette Smoke Exposure. FASEB J. 32 (4), 1880–1890. 10.1096/fj.201700571R 29196503 PMC5893175

[B97] Moore J. B. June C. H. (2020). Cytokine Release Syndrome in Severe COVID-19. Science 368 (6490), 473–474. 10.1126/science.abb8925 32303591

[B98] Morgan J. E. Prola A. Mariot V. Pini V. Meng J. Hourde C. (2018). Necroptosis Mediates Myofibre Death in Dystrophin-Deficient Mice. Nat. Commun. 9 (1), 3655. 10.1038/s41467-018-06057-9 30194302 PMC6128848

[B99] Muendlein H. I. Connolly W. M. Magri Z. Smirnova I. Ilyukha V. Gautam A. (2021). ZBP1 Promotes LPS-Induced Cell Death and IL-1β Release via RHIM-Mediated Interactions with RIPK1. Nat. Commun. 12 (1), 86. 10.1038/s41467-020-20357-z 33397971 PMC7782486

[B100] Nakamura H. Kinjo T. Arakaki W. Miyagi K. Tateyama M. Fujita J. (2020). Serum Levels of Receptor-Interacting Protein Kinase-3 in Patients with COVID-19. Crit. Care 24 (1), 484. 10.1186/s13054-020-03209-6 32753065 PMC7399594

[B101] Newton K. Dugger D. L. Maltzman A. Greve J. M. Hedehus M. Martin-McNulty B. (2016a). RIPK3 Deficiency or Catalytically Inactive RIPK1 Provides Greater Benefit Than MLKL Deficiency in Mouse Models of Inflammation and Tissue Injury. Cell Death Differ 23 (9), 1565–1576. 10.1038/cdd.2016.46 27177019 PMC5072432

[B102] Newton K. Dugger D. L. Wickliffe K. E. Kapoor N. de Almagro M. C. Vucic D. (2014). Activity of protein kinase RIPK3 determines whether cells die by necroptosis or apoptosis. Science 343 (6177), 1357–1360. 10.1126/science.1249361 24557836

[B103] Newton K. Wickliffe K. E. Dugger D. L. Maltzman A. Roose-Girma M. Dohse M. (2019). Cleavage of RIPK1 by Caspase-8 Is Crucial for Limiting Apoptosis and Necroptosis. Nature 574 (7778), 428–431. 10.1038/s41586-019-1548-x 31511692

[B104] Newton K. Wickliffe K. E. Maltzman A. Dugger D. L. Strasser A. Pham V. C. (2016b). RIPK1 Inhibits ZBP1-Driven Necroptosis during Development. Nature 540 (7631), 129–133. 10.1038/nature20559 27819682

[B105] Nogusa S. Thapa R. J. Dillon C. P. Liedmann S. Oguin T. H. 3rd Ingram J. P. (2016). RIPK3 Activates Parallel Pathways of MLKL-Driven Necroptosis and FADD-Mediated Apoptosis to Protect against Influenza A Virus. Cell Host Microbe 20 (1), 13–24. 10.1016/j.chom.2016.05.011 27321907 PMC5026823

[B106] Nyiramana M. M. Cho S. B. Kim E. J. Kim M. J. Ryu J. H. Nam H. J. (2020). Sea Hare Hydrolysate-Induced Reduction of Human Non-small Cell Lung Cancer Cell Growth through Regulation of Macrophage Polarization and Non-apoptotic Regulated Cell Death Pathways. Cancers (Basel) 12 (3), 726. 10.3390/cancers12030726 PMC7140097 32204484

[B107] Okano M. Kariya S. Ohta N. Imoto Y. Fujieda S. Nishizaki K. (2015). Association and Management of Eosinophilic Inflammation in Upper and Lower Airways. Allergol. Int. 64 (2), 131–138. 10.1016/j.alit.2015.01.004 25838087

[B108] Pan L. Yao D. C. Yu Y. Z. Chen B. J. Li S. J. Hu G. H. (2016a). Activation of Necroptosis in a Rat Model of Acute Respiratory Distress Syndrome Induced by Oleic Acid. Sheng Li Xue Bao 68 (5), 661–668. 27778032

[B109] Pan L. Yao D. C. Yu Y. Z. Li S. J. Chen B. J. Hu G. H. (2016b). Necrostatin-1 Protects against Oleic Acid-Induced Acute Respiratory Distress Syndrome in Rats. Biochem. Biophys. Res. Commun. 478 (4), 1602–1608. 10.1016/j.bbrc.2016.08.163 27586277

[B110] Park J. E. Lee J. H. Lee S. Y. Hong M. J. Choi J. E. Park S. (2020). Expression of Key Regulatory Genes in Necroptosis and its Effect on the Prognosis in Non-small Cell Lung Cancer. J. Cancer 11 (18), 5503–5510. 10.7150/jca.46172 32742497 PMC7391199

[B111] Park J. H. Jung K. H. Kim S. J. Yoon Y. C. Yan H. H. Fang Z. (2019). HS-173 as a Novel Inducer of RIP3-dependent Necroptosis in Lung Cancer. Cancer Lett. 444, 94–104. 10.1016/j.canlet.2018.12.006 30583075

[B112] Pasparakis M. Vandenabeele P. (2015). Necroptosis and its Role in Inflammation. Nature 517 (7534), 311–320. 10.1038/nature14191 25592536

[B113] Paudel S. Ghimire L. Jin L. Baral P. Cai S. Jeyaseelan S. (2019). NLRC4 Suppresses IL-17A-mediated Neutrophil-dependent Host Defense through Upregulation of IL-18 and Induction of Necroptosis during Gram-Positive Pneumonia. Mucosal Immunol. 12 (1), 247–257. 10.1038/s41385-018-0088-2 30279514 PMC6301100

[B114] Pehote G. Vij N. (2020). Autophagy Augmentation to Alleviate Immune Response Dysfunction, and Resolve Respiratory and COVID-19 Exacerbations. Cells 9 (9), 1952. 10.3390/cells9091952 PMC7565665 32847034

[B115] Peixoto M. S. de Oliveira Galvão M. F. Batistuzzo de Medeiros S. R. (2017). Cell Death Pathways of Particulate Matter Toxicity. Chemosphere 188, 32–48. 10.1016/j.chemosphere.2017.08.076 28865791

[B116] Petanidis S. Domvri K. Porpodis K. Anestakis D. Freitag L. Hohenforst-Schmidt W. (2020). Inhibition of Kras-Derived Exosomes Downregulates Immunosuppressive BACH2/GATA-3 Expression via RIP-3 Dependent Necroptosis and miR-146/miR-210 Modulation. Biomed. Pharmacother. 122, 109461. 10.1016/j.biopha.2019.109461 31918262

[B117] Pouwels S. D. Zijlstra G. J. van der Toorn M. Hesse L. Gras R. Ten Hacken N. H. (2016). Cigarette Smoke-Induced Necroptosis and DAMP Release Trigger Neutrophilic Airway Inflammation in Mice. Am. J. Physiol. Lung Cell Mol Physiol 310 (4), L377–L386. 10.1152/ajplung.00174.2015 26719146

[B118] Qin C. Sai X. Y. Qian X. F. Wu Y. Zou L. F. Wang H. M. (2019). Close Relationship between cIAP2 and Human ARDS Induced by Severe H7N9 Infection. Biomed. Res. Int. 2019, 2121357. 10.1155/2019/2121357 31080811 PMC6475567

[B119] Qing D. Y. Conegliano D. Shashaty M. G. Seo J. Reilly J. P. Worthen G. S. (2014). Red Blood Cells Induce Necroptosis of Lung Endothelial Cells and Increase Susceptibility to Lung Inflammation. Am. J. Respir. Crit. Care Med. 190 (11), 1243–1254. 10.1164/rccm.201406-1095OC 25329368 PMC4315814

[B120] Rabe K. F. Watz H. (2017). Chronic Obstructive Pulmonary Disease. Lancet 389 (10082), 1931–1940. 10.1016/s0140-6736(17)31222-9 28513453

[B121] Rabinovitch M. Guignabert C. Humbert M. Nicolls M. R. (2014). Inflammation and Immunity in the Pathogenesis of Pulmonary Arterial Hypertension. Circ. Res. 115 (1), 165–175. 10.1161/CIRCRESAHA.113.301141 24951765 PMC4097142

[B122] Radonjic-Hoesli S. Wang X. de Graauw E. Stoeckle C. Styp-Rekowska B. Hlushchuk R. (2017). Adhesion-induced Eosinophil Cytolysis Requires the Receptor-Interacting Protein Kinase 3 (RIPK3)-Mixed Lineage Kinase-like (MLKL) Signaling Pathway, Which Is Counterregulated by Autophagy. J. Allergy Clin. Immunol. 140 (6), 1632–1642. 10.1016/j.jaci.2017.01.044 28412393

[B123] Richeldi L. Collard H. R. Jones M. G. (2017). Idiopathic Pulmonary Fibrosis. Lancet 389 (10082), 1941–1952. 10.1016/s0140-6736(17)30866-8 28365056

[B124] Riegler A. N. Brissac T. Gonzalez-Juarbe N. Orihuela C. J. (2019). Necroptotic Cell Death Promotes Adaptive Immunity against Colonizing Pneumococci. Front Immunol. 10, 615. 10.3389/fimmu.2019.00615 31019504 PMC6459137

[B125] Roca F. J. Ramakrishnan L. (2013). TNF Dually Mediates Resistance and Susceptibility to Mycobacteria via Mitochondrial Reactive Oxygen Species. Cell 153 (3), 521–534. 10.1016/j.cell.2013.03.022 23582643 PMC3790588

[B126] Roy M. P. (2019). COPD and Indoor Air Pollution. BMJ 367, l6167. 10.1136/bmj.l6167 31662291

[B127] Ryter S. W. Chen Z. H. Kim H. P. Choi A. M. (2009). Autophagy in Chronic Obstructive Pulmonary Disease: Homeostatic or Pathogenic Mechanism? Autophagy 5 (2), 235–237. 10.4161/auto.5.2.7495 19066468

[B128] Saddoughi S. A. Gencer S. Peterson Y. K. Ward K. E. Mukhopadhyay A. Oaks J. (2013). Sphingosine Analogue Drug FTY720 Targets I2PP2A/SET and Mediates Lung Tumour Suppression via Activation of PP2A-RIPK1-dependent Necroptosis. EMBO Mol. Med. 5 (1), 105–121. 10.1002/emmm.201201283 23180565 PMC3569657

[B129] Samir P. Malireddi R. K. S. Kanneganti T. D. (2020). The PANoptosome: A Deadly Protein Complex Driving Pyroptosis, Apoptosis, and Necroptosis (PANoptosis). Front Cell Infect Microbiol 10, 238. 10.3389/fcimb.2020.00238 32582562 PMC7283380

[B130] Scaffidi P. Misteli T. Bianchi M. E. (2002). Release of Chromatin Protein HMGB1 by Necrotic Cells Triggers Inflammation. Nature 418 (6894), 191–195. 10.1038/nature00858 12110890

[B131] Schuliga M. Read J. Knight D. A. (2021). Ageing Mechanisms that Contribute to Tissue Remodeling in Lung Disease. Ageing Res. Rev. 70, 101405. 10.1016/j.arr.2021.101405 34242806

[B132] Schwarzer R. Jiao H. Wachsmuth L. Tresch A. Pasparakis M. (2020). FADD and Caspase-8 Regulate Gut Homeostasis and Inflammation by Controlling MLKL- and GSDMD-Mediated Death of Intestinal Epithelial Cells. Immunity 52 (6), 978. 10.1016/j.immuni.2020.04.002 32362323

[B133] Seehawer M. Heinzmann F. D'Artista L. Harbig J. Roux P. F. Hoenicke L. (2018). Necroptosis Microenvironment Directs Lineage Commitment in Liver Cancer. Nature 562 (7725), 69–75. 10.1038/s41586-018-0519-y 30209397 PMC8111790

[B134] Seifert L. Werba G. Tiwari S. Giao Ly N. N. Alothman S. Alqunaibit D. (2016). The Necrosome Promotes Pancreatic Oncogenesis via CXCL1 and Mincle-Induced Immune Suppression. Nature 532 (7598), 245–249. 10.1038/nature17403 27049944 PMC4833566

[B135] Shan B. Pan H. Najafov A. Yuan J. (2018). Necroptosis in Development and Diseases. Genes Dev. 32 (5-6), 327–340. 10.1101/gad.312561.118 29593066 PMC5900707

[B136] Shashaty M. G. S. Reilly J. P. Faust H. E. Forker C. M. Ittner C. A. G. Zhang P. X. (2019). Plasma Receptor Interacting Protein Kinase-3 Levels Are Associated with Acute Respiratory Distress Syndrome in Sepsis and Trauma: a Cohort Study. Crit. Care 23 (1), 235. 10.1186/s13054-019-2482-x 31253195 PMC6599265

[B137] Shi C. Kim T. Steiger S. Mulay S. R. Klinkhammer B. M. Bäuerle T. (2020). Crystal Clots as Therapeutic Target in Cholesterol Crystal Embolism. Circ. Res. 126 (8), e37–e52. 10.1161/CIRCRESAHA.119.315625 32089086

[B138] Shlomovitz I. Erlich Z. Speir M. Zargarian S. Baram N. Engler M. (2019). Necroptosis Directly Induces the Release of Full-Length Biologically Active IL-33 *In Vitro* and in an Inflammatory Disease Model. FEBS J. 286 (3), 507–522. 10.1111/febs.14738 30576068

[B139] Siempos II Ma K. C. Imamura M. Baron R. M. Fredenburgh L. E. Huh J. W. (2018). RIPK3 Mediates Pathogenesis of Experimental Ventilator-Induced Lung Injury. JCI Insight 3 (9). 10.1172/jci.insight.97102 PMC6012515 29720570

[B140] Simpson J. Loh Z. Ullah M. A. Lynch J. P. Werder R. B. Collinson N. (2020). Respiratory Syncytial Virus Infection Promotes Necroptosis and HMGB1 Release by Airway Epithelial Cells. Am. J. Respir. Crit. Care Med. 201 (11), 1358–1371. 10.1164/rccm.201906-1149OC 32105156

[B141] Strilic B. Yang L. Albarrán-Juárez J. Wachsmuth L. Han K. Müller U. C. (2016). Tumour-cell-induced Endothelial Cell Necroptosis via Death Receptor 6 Promotes Metastasis. Nature 536 (7615), 215–218. 10.1038/nature19076 27487218

[B142] Stutz M. D. Ojaimi S. Allison C. Preston S. Arandjelovic P. Hildebrand J. M. (2018a). Necroptotic Signaling Is Primed in Mycobacterium Tuberculosis-Infected Macrophages, but its Pathophysiological Consequence in Disease Is Restricted. Cell Death Differ 25 (5), 951–965. 10.1038/s41418-017-0031-1 29229989 PMC5943269

[B143] Stutz M. D. Ojaimi S. Ebert G. Pellegrini M. (2018b). Is Receptor-Interacting Protein Kinase 3 a Viable Therapeutic Target for Mycobacterium tuberculosis Infection? Front Immunol. 9, 1178. 10.3389/fimmu.2018.01178 29892302 PMC5985376

[B144] Su Z. Yang Z. Xie L. DeWitt J. P. Chen Y. (2016). Cancer Therapy in the Necroptosis Era. Cell Death Differ 23 (5), 748–756. 10.1038/cdd.2016.8 26915291 PMC4832112

[B145] Sun T. Ding W. Xu T. Ao X. Yu T. Li M. (2019a). Parkin Regulates Programmed Necrosis and Myocardial Ischemia/Reperfusion Injury by Targeting Cyclophilin-D. Antioxid. Redox Signal 31 (16), 1177–1193. 10.1089/ars.2019.7734 31456416

[B146] Sun W. Bao J. Lin W. Gao H. Zhao W. Zhang Q. (2016). 2-Methoxy-6-acetyl-7-methyljuglone (MAM), a Natural Naphthoquinone, Induces NO-dependent Apoptosis and Necroptosis by H2O2-dependent JNK Activation in Cancer Cells. Free Radic. Biol. Med. 92, 61–77. 10.1016/j.freeradbiomed.2016.01.014 26802903

[B147] Sun W. Yu J. Gao H. Wu X. Wang S. Hou Y. (2019b). Inhibition of Lung Cancer by 2-Methoxy-6-Acetyl-7-Methyljuglone through Induction of Necroptosis by Targeting Receptor-Interacting Protein 1. Antioxid. Redox Signal 31 (2), 93–108. 10.1089/ars.2017.7376 30556404

[B148] Takezaki A. Tsukumo S. I. Setoguchi Y. Ledford J. G. Goto H. Hosomichi K. (2019). A Homozygous SFTPA1 Mutation Drives Necroptosis of Type II Alveolar Epithelial Cells in Patients with Idiopathic Pulmonary Fibrosis. J. Exp. Med. 216 (12), 2724–2735. 10.1084/jem.20182351 31601679 PMC6888986

[B149] Tang X. Li Y. Liu L. Guo R. Zhang P. Zhang Y. (2020). Sirtuin 3 Induces Apoptosis and Necroptosis by Regulating Mutant P53 Expression in Small-cell L-ung C-ancer. Oncol. Rep. 43 (2), 591–600. 10.3892/or.2019.7439 31894331

[B150] Thapa R. J. Ingram J. P. Ragan K. B. Nogusa S. Boyd D. F. Benitez A. A. (2016). DAI Senses Influenza A Virus Genomic RNA and Activates RIPK3-dependent Cell Death. Cell Host Microbe 20 (5), 674–681. 10.1016/j.chom.2016.09.014 27746097 PMC5687825

[B151] Tompson D. J. Davies C. Scott N. E. Cannons E. P. Kostapanos M. Gross A. S. (2021). Comparison of the Pharmacokinetics of RIPK1 Inhibitor GSK2982772 in Healthy Western and Japanese Subjects. Eur. J. Drug Metab. Pharmacokinet. 46 (1), 71–83. 10.1007/s13318-020-00652-2 33165774 PMC7811991

[B152] Upton J. W. Kaiser W. J. Mocarski E. S. (2019). DAI/ZBP1/DLM-1 Complexes with RIP3 to Mediate Virus-Induced Programmed Necrosis that Is Targeted by Murine Cytomegalovirus vIRA. Cell Host Microbe 26 (4), 564. 10.1016/j.chom.2019.09.004 31600504

[B153] Vanden Berghe T. Hassannia B. Vandenabeele P. (2016). An Outline of Necrosome Triggers. Cell Mol Life Sci 73 (11-12), 2137–2152. 10.1007/s00018-016-2189-y 27052312 PMC4887535

[B154] Vanden Berghe T. Linkermann A. Jouan-Lanhouet S. Walczak H. Vandenabeele P. (2014). Regulated Necrosis: the Expanding Network of Non-apoptotic Cell Death Pathways. Nat. Rev. Mol. Cell Biol 15 (2), 135–147. 10.1038/nrm3737 24452471

[B155] Vishnupriya S. Priya Dharshini L. C. Sakthivel K. M. Rasmi R. R. (2020). Autophagy Markers as Mediators of Lung Injury-Implication for Therapeutic Intervention. Life Sci. 260, 118308. 10.1016/j.lfs.2020.118308 32828942 PMC7442051

[B156] Vitner E. B. Salomon R. Farfel-Becker T. Meshcheriakova A. Ali M. Klein A. D. (2014). RIPK3 as a Potential Therapeutic Target for Gaucher's Disease. Nat. Med. 20 (2), 204–208. 10.1038/nm.3449 24441827

[B157] Wang H. Sun L. Su L. Rizo J. Liu L. Wang L. F. (2014a). Mixed Lineage Kinase Domain-like Protein MLKL Causes Necrotic Membrane Disruption upon Phosphorylation by RIP3. Mol. Cell 54 (1), 133–146. 10.1016/j.molcel.2014.03.003 24703947

[B158] Wang H. H. Wu Z. Q. Qian D. Zaorsky N. G. Qiu M. H. Cheng J. J. (2018a). Ablative Hypofractionated Radiation Therapy Enhances Non-small Cell Lung Cancer Cell Killing via Preferential Stimulation of Necroptosis *In Vitro* and *In Vivo* . Int. J. Radiat. Oncol. Biol. Phys. 101 (1), 49–62. 10.1016/j.ijrobp.2018.01.036 29619976

[B159] Wang L. Wang T. Li H. Liu Q. Zhang Z. Xie W. (2016). Receptor Interacting Protein 3-Mediated Necroptosis Promotes Lipopolysaccharide-Induced Inflammation and Acute Respiratory Distress Syndrome in Mice. PLoS One 11 (5), e0155723. 10.1371/journal.pone.0155723 27195494 PMC4873150

[B160] Wang M. Zhong D. Dong P. Song Y. (2018b). Blocking CXCR1/2 Contributes to Amelioration of Lipopolysaccharide‐induced Sepsis by Downregulating Substance P. J. Cell Biochem 120, 2007–2014. 10.1002/jcb.27507 30160797

[B161] Wang Q. Wang P. Zhang L. Tessema M. Bai L. Xu X. (2020a). Epigenetic Regulation of RIP3 Suppresses Necroptosis and Increases Resistance to Chemotherapy in NonSmall Cell Lung Cancer. Transl Oncol. 13 (2), 372–382. 10.1016/j.tranon.2019.11.011 31887632 PMC6938879

[B162] Wang R. Li H. Wu J. Cai Z. Y. Li B. Ni H. (2020b). Gut Stem Cell Necroptosis by Genome Instability Triggers Bowel Inflammation. Nature 580 (7803), 386–390. 10.1038/s41586-020-2127-x 32296174

[B163] Wang X. Li Y. Liu S. Yu X. Li L. Shi C. (2014b). Direct Activation of RIP3/MLKL-dependent Necrosis by Herpes Simplex Virus 1 (HSV-1) Protein ICP6 Triggers Host Antiviral Defense. Proc. Natl. Acad. Sci. U S A. 111 (43), 15438–15443. 10.1073/pnas.1412767111 25316792 PMC4217423

[B164] Wang X. O'Brien M. E. Yu J. Xu C. Zhang Q. Lu S. (2019a). Prolonged Cold Ischemia Induces Necroptotic Cell Death in Ischemia-Reperfusion Injury and Contributes to Primary Graft Dysfunction after Lung Transplantation. Am. J. Respir. Cell Mol Biol 61 (2), 244–256. 10.1165/rcmb.2018-0207OC 30742487 PMC6670033

[B165] Wang Y. Hao Q. Florence J. M. Jung B. G. Kurdowska A. K. Samten B. (2019b). Influenza Virus Infection Induces ZBP1 Expression and Necroptosis in Mouse Lungs. Front Cell Infect Microbiol 9, 286. 10.3389/fcimb.2019.00286 31440477 PMC6694206

[B166] Wang Y. Wang X. K. Wu P. P. Wang Y. Ren L. Y. Xu A. H. (2020c). Necroptosis Mediates Cigarette Smoke-Induced Inflammatory Responses in Macrophages. Int. J. Chron. Obstruct Pulmon Dis. 15, 1093–1101. 10.2147/COPD.S233506 32546997 PMC7244448

[B167] Wang Y. Zhou J. S. Xu X. C. Li Z. Y. Chen H. P. Ying S. M. (2018c). Endoplasmic Reticulum Chaperone GRP78 Mediates Cigarette Smoke-Induced Necroptosis and Injury in Bronchial Epithelium. Int. J. Chron. Obstruct Pulmon Dis. 13, 571–581. 10.2147/COPD.S150633 29445274 PMC5810534

[B168] Wark P. A. Gibson P. G. (2003). Clinical Usefulness of Inflammatory Markers in Asthma. Am. J. Respir. Med. 2 (1), 11–19. 10.1007/BF03256635 14720018

[B169] Wegner K. W. Saleh D. Degterev A. (2017). Complex Pathologic Roles of RIPK1 and RIPK3: Moving beyond Necroptosis. Trends Pharmacol. Sci. 38 (3), 202–225. 10.1016/j.tips.2016.12.005 28126382 PMC5325808

[B170] Wei R. Xu L. W. Liu J. Li Y. Zhang P. Shan B. (2017). SPATA2 Regulates the Activation of RIPK1 by Modulating Linear Ubiquitination. Genes Dev. 31 (11), 1162–1176. 10.1101/gad.299776.117 28701375 PMC5538438

[B171] Weisel K. Berger S. Papp K. Maari C. Krueger J. G. Scott N. (2020). Response to Inhibition of Receptor-Interacting Protein Kinase 1 (RIPK1) in Active Plaque Psoriasis: A Randomized Placebo-Controlled Study. Clin. Pharmacol. Ther. 108 (4), 808–816. 10.1002/cpt.1852 32301501 PMC7540322

[B172] Weisel K. Berger S. Thorn K. Taylor P. C. Peterfy C. Siddall H. (2021a). A Randomized, Placebo-Controlled Experimental Medicine Study of RIPK1 Inhibitor GSK2982772 in Patients with Moderate to Severe Rheumatoid Arthritis. Arthritis Res. Ther. 23 (1), 85. 10.1186/s13075-021-02468-0 33726834 PMC7962407

[B173] Weisel K. Scott N. Berger S. Wang S. Brown K. Powell M. (2021b). A Randomised, Placebo-Controlled Study of RIPK1 Inhibitor GSK2982772 in Patients with Active Ulcerative Colitis. BMJ Open Gastroenterol. 8 (1), e000680. 10.1136/bmjgast-2021-000680 PMC8365785 34389633

[B174] Weisel K. Scott N. E. Tompson D. J. Votta B. J. Madhavan S. Povey K. (2017). Randomized Clinical Study of Safety, Pharmacokinetics, and Pharmacodynamics of RIPK1 Inhibitor GSK2982772 in Healthy Volunteers. Pharmacol. Res. Perspect. 5 (6), e00365. 10.1002/prp2.365 PMC5723699 29226626

[B175] Xiao G. Zhuang W. Wang T. Lian G. Luo L. Ye C. (2020). Transcriptomic Analysis Identifies Toll-like and Nod-like Pathways and Necroptosis in Pulmonary Arterial Hypertension. J. Cell Mol Med 24 (19), 11409–11421. 10.1111/jcmm.15745 32860486 PMC7576255

[B176] Xu F. Luo M. He L. Cao Y. Li W. Ying S. (2018). Necroptosis Contributes to Urban Particulate Matter-Induced Airway Epithelial Injury. Cell Physiol Biochem 46 (2), 699–712. 10.1159/000488726 29621753

[B177] Yang L. Joseph S. Sun T. Hoffmann J. Thevissen S. Offermanns S. (2019). TAK1 Regulates Endothelial Cell Necroptosis and Tumor Metastasis. Cell Death Differ 26 (10), 1987–1997. 10.1038/s41418-018-0271-8 30683914 PMC6748133

[B178] Yokohori N. Aoshiba K. Nagai A. (2004). Increased Levels of Cell Death and Proliferation in Alveolar wall Cells in Patients with Pulmonary Emphysema. Chest 125 (2), 626–632. 10.1378/chest.125.2.626 14769747

[B179] Yoon S. Kovalenko A. Bogdanov K. Wallach D. (2017). MLKL, the Protein that Mediates Necroptosis, Also Regulates Endosomal Trafficking and Extracellular Vesicle Generation. Immunity 47 (1), 51–e7. 10.1016/j.immuni.2017.06.001 28666573

[B180] Yu W. N. Lai Y. J. Ma J. W. Ho C. T. Hung S. W. Chen Y. H. (2019). Citronellol Induces Necroptosis of Human Lung Cancer Cells via TNF-α Pathway and Reactive Oxygen Species Accumulation. In Vivo 33 (4), 1193–1201. 10.21873/invivo.11590 31280209 PMC6689369

[B181] Yu X. Mao M. Liu X. Shen T. Li T. Yu H. (2020). A Cytosolic Heat Shock Protein 90 and Co-chaperone P23 Complex Activates RIPK3/MLKL during Necroptosis of Endothelial Cells in Acute Respiratory Distress Syndrome. J. Mol. Med. (Berl) 98 (4), 569–583. 10.1007/s00109-020-01886-y 32072232

[B182] Yuan J. Amin P. Ofengeim D. (2019). Necroptosis and RIPK1-Mediated Neuroinflammation in CNS Diseases. Nat. Rev. Neurosci. 20 (1), 19–33. 10.1038/s41583-018-0093-1 30467385 PMC6342007

[B183] Zemskova M. McClain N. Niihori M. Varghese M. V. James J. Rafikov R. (2020). Necrosis-Released HMGB1 (High Mobility Group Box 1) in the Progressive Pulmonary Arterial Hypertension Associated with Male Sex. Hypertension 76 (6), 1787–1799. 10.1161/HYPERTENSIONAHA.120.16118 33012199 PMC7666015

[B184] Zhan C. Huang M. Yang X. Hou J. (2021). MLKL: Functions beyond Serving as the Executioner of Necroptosis. Theranostics 11 (10), 4759–4769. 10.7150/thno.54072 33754026 PMC7978304

[B185] Zhang H. Liu Q. Kong L. Xu S. (2019b). Mucin 1 Downregulation Impairs the Anti-necroptotic Effects of Glucocorticoids in Human Bronchial Epithelial Cells. Life Sci. 221, 168–177. 10.1016/j.lfs.2019.02.013 30738043

[B186] Zhang H. Ji J. Liu Q. Xu S. (2019a). MUC1 Downregulation Promotes TNF‐α‐induced Necroptosis in Human Bronchial Epithelial Cells via Regulation of the RIPK1/RIPK3 Pathway. J. Cell Physiol 234, 15080–15088. 10.1002/jcp.28148 PMC6590293 30666647

[B187] Zhang T. Yin C. Boyd D. F. Quarato G. Ingram J. P. Shubina M. (2020). Influenza Virus Z-RNAs Induce ZBP1-Mediated Necroptosis. Cell 180 (6), 1115. 10.1016/j.cell.2020.02.050 32200799 PMC7153753

[B188] Zhang T. Zhang Y. Cui M. Jin L. Wang Y. Lv F. (2016). CaMKII Is a RIP3 Substrate Mediating Ischemia- and Oxidative Stress-Induced Myocardial Necroptosis. Nat. Med. 22 (2), 175–182. 10.1038/nm.4017 26726877

[B189] Zhao H. Chen Q. Huang H. Suen K. C. Alam A. Cui J. (2019a). Osteopontin Mediates Necroptosis in Lung Injury after Transplantation of Ischaemic Renal Allografts in Rats. Br. J. Anaesth. 123 (4), 519–530. 10.1016/j.bja.2019.05.041 31262508

[B190] Zhao H. Ning J. Lemaire A. Koumpa F. S. Sun J. J. Fung A. (2015). Necroptosis and Parthanatos Are Involved in Remote Lung Injury after Receiving Ischemic Renal Allografts in Rats. Kidney Int. 87 (4), 738–748. 10.1038/ki.2014.388 25517913

[B191] Zhao X. Khan N. Gan H. Tzelepis F. Nishimura T. Park S. Y. (2017). Bcl-xL Mediates RIPK3-dependent Necrosis in M. Tuberculosis-Infected Macrophages. Mucosal Immunol. 10 (6), 1553–1568. 10.1038/mi.2017.12 28401933 PMC5638669

[B192] Zhao Y. Zhang H. Yang X. Zhang Y. Feng S. Yan X. (2019b). Fine Particulate Matter (PM2.5) Enhances Airway Hyperresponsiveness (AHR) by Inducing Necroptosis in BALB/c Mice. Environ. Toxicol. Pharmacol. 68, 155–163. 10.1016/j.etap.2019.03.013 30986632

[B193] Zheng M. Kanneganti T. D. (2020). The Regulation of the ZBP1-NLRP3 Inflammasome and its Implications in Pyroptosis, Apoptosis, and Necroptosis (PANoptosis). Immunol. Rev. 297 (1), 26–38. 10.1111/imr.12909 32729116 PMC7811275

[B194] Zhou L. L. Wang M. Liu F. Lu Y. Z. Song L. J. Xiong L. (2019). Cigarette Smoking Aggravates Bleomycin-Induced Experimental Pulmonary Fibrosis. Toxicol. Lett. 303, 1–8. 10.1016/j.toxlet.2018.12.008 30572104

[B195] Zhou Y. Niu C. Ma B. Xue X. Li Z. Chen Z. (2018). Inhibiting PSMα-Induced Neutrophil Necroptosis Protects Mice with MRSA Pneumonia by Blocking the Agr System. Cell Death Dis 9 (3), 362. 10.1038/s41419-018-0398-z 29500427 PMC5834619

